# Progress of Enzymatic and Non-Enzymatic Electrochemical Glucose Biosensor Based on Nanomaterial-Modified Electrode

**DOI:** 10.3390/bios12121136

**Published:** 2022-12-06

**Authors:** Noorhashimah Mohamad Nor, Nur Syafinaz Ridhuan, Khairunisak Abdul Razak

**Affiliations:** 1 School of Materials and Mineral Resources Engineering, Universiti Sains Malaysia, Nibong Tebal 14300, Pulau Pinang, Malaysia; 2 NanoBiotechnology Research & Innovation (NanoBRI), Institute for Research in Molecular Medicine, Universiti Sains Malaysia, Gelugor 11800, Pulau Pinang, Malaysia

**Keywords:** glucose detection, electrochemical sensor, enzyme immobilization, glucose oxidation, metal and metal oxide nanomaterials

## Abstract

This review covers the progress of nanomaterial-modified electrodes for enzymatic and non-enzymatic glucose biosensors. Fundamental insights into glucose biosensor components and the crucial factors controlling the electrochemical performance of glucose biosensors are discussed in detail. The metal, metal oxide, and hybrid/composite nanomaterial fabrication strategies for the modification of electrodes, mechanism of detection, and significance of the nanomaterials toward the electrochemical performance of enzymatic and non-enzymatic glucose biosensors are compared and comprehensively reviewed. This review aims to provide readers with an overview and underlying concept of producing a reliable, stable, cost-effective, and excellent electrochemical performance of a glucose biosensor.

## 1. Introduction

Diabetes mellitus is a chronic disease that occurs when the pancreas fails to produce sufficient insulin to regulate blood sugar or when the body is unable to use the insulin produced effectively. If diabetes is not well treated, a number of other health problems may follow, such as eye complications, neuropathy, foot complications, kidney disease, hypertension, stroke, hyperglycemic nonketonic syndrome, gastroparesis, heart disease, and mental health disorders; it may also affect pregnancy [1,2]. Tight control of diabetes is critical to prevent or slow down the progress of diabetes complications. A normal blood glucose level in human serum before a meal is around 4–6 mM (70–110 mg/dL) and <7.8 mM (<140 mg/dL) after 2 h of mealtime [3,4]. In diabetic patients, the normal glucose concentration in serum is between 5.6 and 6.9 mM (100–125 mg/dL) before mealtime and 7.8 and 11 mM (140–199 mg/dL) after 2 h of mealtime [5]. For efficient therapy and to prevent any hyperglycemia or hypoglycemia, regular monitoring of physiological blood glucose levels is essential.

Clark and Lyons [6] were the first researchers who reported on glucose quantification by employing a dialysis membrane on the oxygen electrode surface based on glucose oxidase (GOx) entrapment via potentiometric measurements. The glucose concentration was analyzed based on the reduction of dissolved oxygen [2]. Since then, research on glucose biosensors has been conducted employing conductometric, impedimetric, potentiometric, and amperometric techniques based on the GOx enzyme, which catalyzes the oxidation of glucose into gluconic acid [7,8,9,10].

At present, the common method of blood glucose monitoring is invasive, which involves finger pricking, collecting a drop of blood on top of the glucose test strip, and analyzing the results by using a glucose meter. The blood sample undergoes an enzymatic chemical reaction at the test strip, followed by electrochemical detection with a glucose meter. Several commercial glucose test strips are available in the market for analysis of blood glucose levels, but these strips have varied performance. Normally, commercial glucose test strips have a glucose detection linearity of 0.5–33 mM and require 0.3–2 µL of blood and 4–5 s of assay time. The commercial glucose strip is able to maintain its stability at 4 °C for 35 to 50 days in an open vial container and 18–24 month in a sealed container. The failure time of a commercial glucose strip is determined as the concentration value at the time of measurement out of range by ±15% from the initial concentration value.

Nowadays, extensive efforts toward the non-invasive technique, which allows wearable [11,12], continuous [13,14], and point-of-care [15,16] blood glucose monitoring have drawn interest among researchers and users. Additionally, the applicability of different types of biofluids such as sweat, tear, urine, saliva, and interstitial fluid to replace blood in monitoring glucose levels is being considered [17,18,19]. Rapid and sensitive glucose biosensors are important not only for clinical chemistry, but also for food and industrial quality analysis [20,21,22]. Therefore, the fabrication of glucose biosensors to enhance sensitivity, accuracy, response time, reliability, long lifetime stability, and cost-effectiveness are important.

Electrochemical sensing strategies are versatile and powerful tools in providing real-time and on-site measurement in a variety of areas, including clinical diagnostic, environmental, agricultural, and food monitoring [23,24,25,26]. The electrochemical sensing provides advantages in offering high sensitivity, selectivity, accuracy, and cost effectiveness. Therefore, biosensors with electrochemical monitoring systems dominate the commercial glucometer market. Electrochemical glucose biosensors are widely applied for glucose monitoring due to their unbeatable sensitivity, selectivity, and simplicity. Electrochemical glucose biosensors can be further classified based on the output signal measuring techniques, namely, amperometric (measures the electrical current produced due to a redox reaction), potentiometric (measures the change in electrode potential), and conductometric (measures the change in charge transfer resistance). Amperometric glucose sensors are the most commonly employed glucose biosensors. Electrochemical glucose biosensors commonly comprise a three-electrode system: working, reference, and counter electrodes. Each type of electrode has a specific function. The working electrode is a sensor or transducer responding to the electrochemical reaction. The reference electrode is a steady and well-known electrode potential that is often based on a saturated calomel electrode (SCE) or silver-silver chloride Ag/AgCl electrode. The counter electrode completes the current circuit by providing a current connection in between the electrocatalytic solutions and the working electrode in electrochemical cell. The counter electrode is usually made of an inert material, such as platinum (Pt), gold (Au), graphite, or glassy carbon [27,28]. Among these three electrodes, the sensitivity and specificity of glucose detection are dependent on the working electrode.

Glucose biosensors are classified into two types: enzymatic and non-enzymatic. The enzymatic glucose biosensor is commonly employed because immobilized GOx enzyme provides excellent specificity and sensitivity to the glucose biosensor [29]. The immobilization of the GOx enzyme on the working electrode surface is an important factor to be considered in biosensor fabrication. The deep position of the active redox center of the GOx enzyme makes the electron exchange between GOx enzymes and the electrode surface difficult. The shape of GOx enzymes may shift after immobilization on the surface of the working electrode [30,31]. Another challenge is to prevent GOx enzyme denaturalization and deactivation, which ultimately reduces the lifetime of the biosensor. Therefore, the immobilization of GOx enzymes on the suitable matrix is crucial to maintain the catalytic properties and stability of the enzyme bioactivity.

**Figure 1:**
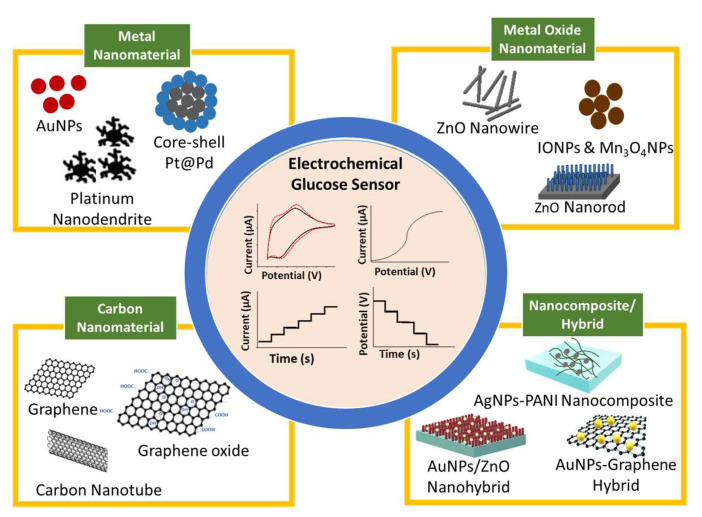
Schematic of nanomaterial-modified electrode for glucose biosensor.

The recent development in glucose biosensors involves modifying the working electrode with nanomaterials, such as metals, metal oxides, and carbon-based nanomaterials, as schematically shown in Figure 1 [32,33,34]. Nanomaterials serve as a matrix to modify the electrode surface and provide a biocompatible area for enzyme immobilization because nanomaterials have a large surface area for reaction activity, good catalytic efficiency, and strong adsorption ability [32,35]. The dependency of enzyme activity on temperature, pH, humidity, and toxic compounds has advanced research on non-enzymatic glucose biosensors [35,36,37]. Non-enzymatic glucose biosensors have excellent sensitivity, good stability, and ease of manufacture, and their current response is directly dependent on the oxidation of glucose on the modified electrode. The main restriction of non-enzymatic glucose biosensors is specificity. Recently, scholars reported high-sensitivity non-enzymatic glucose biosensors based on the modification of electrodes with metal [38,39], metal oxide [17,40], and composite nanomaterials [19,21,41].

Most of the review papers that have been published covered the recent development of enzymatic and nonenzymatic glucose biosensors, and mainly focus on the fabrication strategies and significance of the nanomaterials towards the electrochemical performance of the glucose biosensors [33,42,43,44]. This review paper emphasizes the crucial factors influencing the electrochemical performance of the glucose biosensor, fundamental differences between glucose biosensor generations, biosensing mechanisms of enzymatic and non-enzymatic glucose biosensors, and the fabrication strategies of the modified electrodes. This review comprehensively discusses the progress of nanomaterial-modified electrodes for enzymatic and non-enzymatic electrochemical glucose biosensors primarily from 2010 until recent year of 2022. A comprehensive review on the modification of metal, metal oxide, and carbon-based nanomaterials for enzymatic and non-enzymatic glucose biosensors is discussed in detail. The immobilization strategies, significance of nanomaterials, and morphology of the modified electrode towards the electrochemical performance are comprehensively reviewed. The aim of this paper is to present an exhaustive idea on the fundamental concept and prospect for producing a reliable, stable, and excellent electrochemical performance of the glucose biosensor.

## 2. Generation of Glucose Biosensor

**Figure 2:**
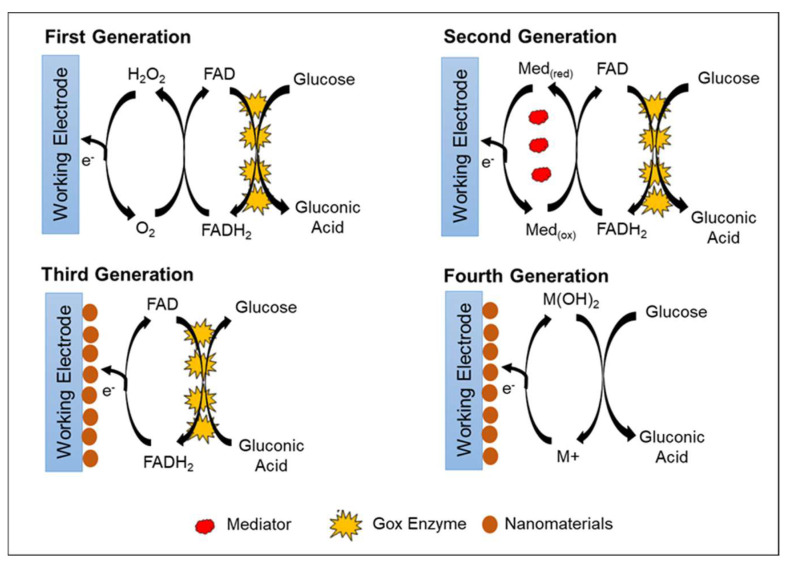
Schematic representation of four generations of glucose biosensors.

In general, there are four primary generations of glucose biosensor, which are classified according to the electron transfer mechanism. Three generations represent the enzymatic glucose biosensor, and one generation represents the non-enzymatic glucose biosensor (Figure 2). The first-generation enzymatic glucose biosensors measure glucose concentration in the analyte sample based on H_2_O_2_ generation or by reduction in oxygen (O_2_) concentration as a natural co-substrate [45]. The immobilized GOx uses molecular O_2_ as an electron acceptor to catalyze the oxidation of D-glucose (C_6_H_10_O_6_) into gluconolactone (C_6_H_12_O_6_), yielding H_2_O_2_ and water as byproducts. As gluconolactone (C_6_H_10_O_6_) hydrolyzes further, gluconic acid (C_6_H_12_O_7_) is created [1,46]. As a catalyst, FAD, which is an active redox center of GOx, plays a role as the initial electron acceptor and is reduced to FADH_2_ in the presence of glucose. The re-oxidation of FADH_2_ with free oxygen generates the oxidized form of the enzyme FAD. In general, the glucose concentration is relative to electrochemical oxidation of the product H_2_O_2_ or electrochemical reduction of O_2_ at the working electrode [47]. The electrons that are transferred are recognized and collected by the counter electrode; thus, the number of glucose molecules present is directly proportional to electron flow [45]. Table 1 lists the advantages and disadvantages of all generations of glucose biosensors.

The advantages of the first-generation glucose biosensor are its simple design and miniaturization of the biosensor [48,49]. However, the first-generation glucose biosensor has limitations in terms of high operation potential needed for the amperometric measurement of H_2_O_2_. This high operation potential may interfere with other electroactive molecules (such as ascorbic acid and uric acids) and some drugs (e.g., acetaminophen) [45]. Another disadvantage is that oxygen deficiency may occur due to the limited oxygen solubility in biological fluids, which causes fluctuations in oxygen tension [50]. The oxygen deficiency then affects the sensor response, narrowing the linearity of the glucose concentration detection ranges.

A variety of techniques have been developed to address the limitations of the first-generation enzymatic glucose biosensor, which are interference from electroactive molecules and oxygen deficiency. Nafion, polyurethane, polycarbonate, or acetate layers were added on the surface of electrode as a selective or protective membrane to minimize the interference toward the electrode and provide mechanical stability to GOx enzyme against denaturalization [51,52]. Electrodes were further modified by co-deposition with metallized materials such as ruthenium and rhodium to lower the operating potentials to approximately 0–0.2 V, which is optimal for preventing electroactivity interference [50,53]. Another approach is to employ oxygen-rich carbon paste enzyme electrodes, which have become an internal source of oxygen due to high oxygen solubility [50].

The second-generation enzymatic glucose biosensor is based on artificial redox mediators in replacing the oxygen-dependent electrode. Mediators are tiny, low-molecular-weight, soluble redox components that act as artificial electron transfer agents. The mediators facilitate electron transport from the FAD active redox center of the enzyme to the working electrode surface [48]. This feature decreases the operational potential of the biosensors at moderate redox potentials, allowing them to avoid the oxidation of other interfering species [54]. Various types of electron mediators that are effective for GOx include ferrocene derivatives, ferricyanide, quinone compounds, conducting polymer salt tetrathiafulvalene-tetracyanoquinodimethane (TTF-TCNQ), transition metal complexes, and phenothiazine [55,56].

During glucose conversion, the electrons produced are collected by the mediator, and the mediator will be reduced to M(red). The mediator releases electrons and transfers the electrons to the electrode at the applied oxidation potential of the mediator. The reduction of the mediator helps facilitate the re-oxidation of the reduced form of GOx (FADH_2_) to GOx (FAD). Further oxidation of the mediator at the electrode surface regenerates M(ox) and two electrons. Thus, the glucose concentration level is proportional to the number of electrons transferred to the counter electrode. With the help of the mediator, measurement of the glucose concentration becomes independent of oxygen partial pressure and can be conducted at a lower potential to minimize interference from electroactive species [57,58].

The weakness of using natural or artificial mediators in glucose biosensor applications is the difficulty to maintain the presence of the mediator near the electrode and enzyme surface [59]. Mediators are small and highly diffusive, so they require additional and complicated methods to secure them near the electrode [60]. Although mediators can react rapidly with the enzyme compared with oxygen, there is also a possibility of dissolved oxygen competing with the mediator, thereby reducing the efficiency of the system and causing a build-up of H_2_O_2_. Another possibility is the reaction between mediator and interference species in the blood, which reduces the accuracy and efficiency of the analytical system [45].

The common oxidation potential of GOx active site (FAD) is around −0.45 V or −0.34 V versus Ag/AgCl [61]. Thus, a suitable mediator applied should have redox potential that is more positive than FAD [62]. Among all electron mediators, ferrocene and its derivatives are commonly applied in the fabrication of electrochemical glucose biosensors. Ferrocene and its derivatives are of interest due to their properties of a wide range of redox potentials, pH independence, rapid electron transfer rate, and high stability in both conditions (oxidized and reduced forms) [48,63].

The problems with mediators, such as poor electron transport, mediator leakage, and poor stability, can be overcome by incorporating polymers and their derivative to the mediator. Polymers improve the mediator biocompatibility, stability, and electrical conductivity, and provide a large surface area. Dendrimers, conducting polymers (polypyrrole and polythiophene), carbon nanotubes (CNTs), chitosan, polyelectrolyte, and polyethylenimine are commonly used [64,65,66]. Jiang et al. [66] reported the use of a ferrocene-modified polyelectrolyte film-coated electrode for amperometric glucose biosensors. The ferrocene group present on the polyelectrolyte skeleton structure acts as a mediator to shuttle electron transfer between FAD-active redox center of GOx enzyme to the working electrode. The modified electrode shows good linearity for glucose detection in the range of 0.2–5 mM.

In third-generation enzymatic glucose biosensors, direct electron transfer between the enzyme and electrode is introduced without the need for natural or synthetic mediators. The FAD-active redox center of the enzyme is covalently or electrochemically linked to the working electrode by nanomaterials. Nanomaterials act as a matrix to enable GOx to be immobilized directly in proximity and facilitate direct electron transfer. Thus, the obtained electrochemical signal is correlated with the glucose concentration [67].

In recent years, efforts to achieve direct electron transfer using various types and sizes of nanomaterials and nanocomposite have been extensively explored due to their excellent physical, chemical, and electronic properties [32,33,68]. Different nanomaterials display various main functions in improving glucose biosensor performance based on their unique properties. However, the basic functions of nanomaterials in biosensors are aiding biomolecule immobilization and labelling, catalysis of electrochemical reactions, increasing the electron transfer rate, and acting as the reactant [32]. The most commonly used nanomaterials are metal [69,70,71], carbon-based [72,73], and metal oxide [74,75,76].

During biosensor fabrication, nanomaterial-modified electrodes have a great potential to adsorb biomolecules and serve as an immobilization support for biomolecules. The direct adsorption of biomolecules onto bulk materials frequently results in denaturation and loss of bioactivity, whereas nanoparticles preserve biomolecule bioactivity [77]. Nanomaterials provide a microenvironment similar to the redox protein in the native system, thereby allowing more freedom for biomolecules to immobilize [78]. Several nanomaterials carry charges via functionalization, providing an electrostatic surface to attach the biomolecules with different charges [77]. The incorporation of suitable surface functional groups on nanoparticles can produce a strong binding of biomolecules with nanoparticles. The high-conductivity properties of nanoparticles allow them to function as a signal-generating probe and a signal amplifier [46]. The third generation of enzymatic glucose biosensors has numerous advantages, including high selectivity and sensitivity toward glucose rather than interfering species such as ascorbic acid and uric acid, a rapid response time, and a low operating potential [79]. Some limitations of the third-generation enzymatic glucose biosensors are enzyme leaching and a good conductivity of nanomaterials to enhance direct electron transfer between the deeply buried FAD-active redox center of the enzyme and the working electrode.

Finally, the fourth generation of glucose biosensors, also known as non-enzymatic glucose biosensors, employs direct electron transfer through electro-oxidation of glucose to gluconic acid at the nanomaterial matrix with strong electrocatalytic activity [80]. In the non-enzymatic glucose biosensor, atoms from nanomaterials act as electrocatalyst in the glucose reaction [81]. Recently, many studies focused on the non-enzymatic glucose biosensor, which employs various types of nanomaterials and nanocomposite materials in the modification of the electrode. However, several issues limit the application of fourth-generation glucose biosensors for commercial use in monitoring patients with diabetes, such as poor selectivity and the requirement for alkaline condition during analysis. Indeed, with a broader understanding of the mechanisms of catalytic properties of nanomaterials, the 3D enzyme mimicking glucose biosensor can be developed.

**Table 1:** The advantages and disadvantages of all generation of glucose biosensor.

Types of Glucose Sensor	Advantages	Disadvantages	Reference
First Generation (Enzymatic)	Simple biosensor design	Need high operating voltage (>1 V) Limit to solubility of oxygen in biological fluid Deactivation of enzyme due to production of H_2_O_2_	[82]
Second Generation (Enzymatic)	Low operating potential (<0.6 V) Mediator aid in electron transfer Less dependence on oxygen presence	Mediator leaching due to small and easy to diffuse properties Competition with dissolved O_2_ Possible to react with interfering species	[66]
Third Generation (Enzymatic)	High selectivity and specificity Nanomaterials facilitate direct electron transfer Low operating potential (<0.6 V)	Requires high conductivity of nanomaterial FAD redox co-factor of enzyme buried deep inside Enzyme leaching	[79]
Fourth Generation (Non-Enzymatic)	High stability Low production cost, as do not use enzyme	Low specificity High interference against interfering species	[35]

## 3. Parameters Controlling Enzymatic and Non-Enzymatic Glucose Biosensors

### 3.1. Glucose

**Figure 3:**
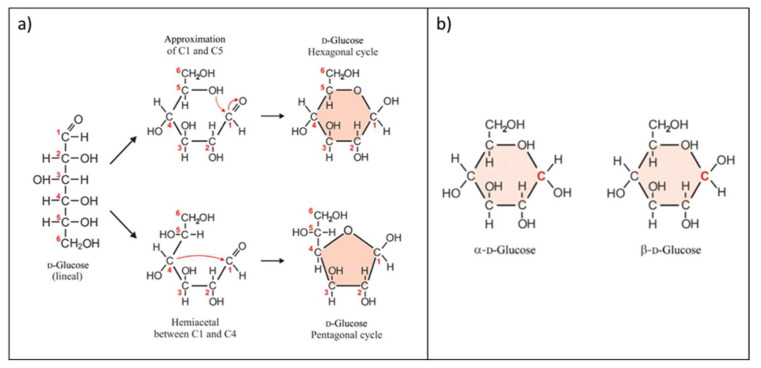
(**a**) Linear form of D-glucose undergoes an intramolecular reaction to form a cyclic hemiacetal; (**b**) different forms of glucose. Reprinted with permission from ref. [85]; Copyright 2017 Elsevier.

Glucose is a monosaccharide containing six carbon atoms and an aldehyde group, sometimes known as an aldohexose with the molecular formula of C_6_H_12_O_6_ [83]. D-glucose, often known as dextrose, is a natural glucose molecule source. The intramolecular interaction between the alcohol group and the aldehyde group of glucose molecules results in the formation of an intramolecular hemiacetal (Figure 3a). As a result of the intramolecular reaction, glucose molecules may exist in an open chain (acyclic) and ring (cyclic) form. Figure 3a shows that the linear form of D-glucose undergoes an intramolecular reaction to form a cyclic hemiacetal. In solid form, glucose is usually present as a monohydrate with a closed pyran ring (dextrose hydrate). In aqueous solution, D-glucose has a tiny open chain and is mostly present as α- or β-glucose, which is typically merged by mutarotation, as illustrated in Figure 3b [84]. Glucose is one of the body’s primary sources of energy in the form of carbohydrates. Our body digests glucose multiple times a day. Glucose is generated by the enzymatic breakdown of glycogen with the help of the pancreas. The process is known as glycogenolysis. Glucose is also called blood sugar, as it circulates in the blood at a concentration of 65–110 mg/dL (4–6 mM).

### 3.2. Enzymes

**Figure 4:**
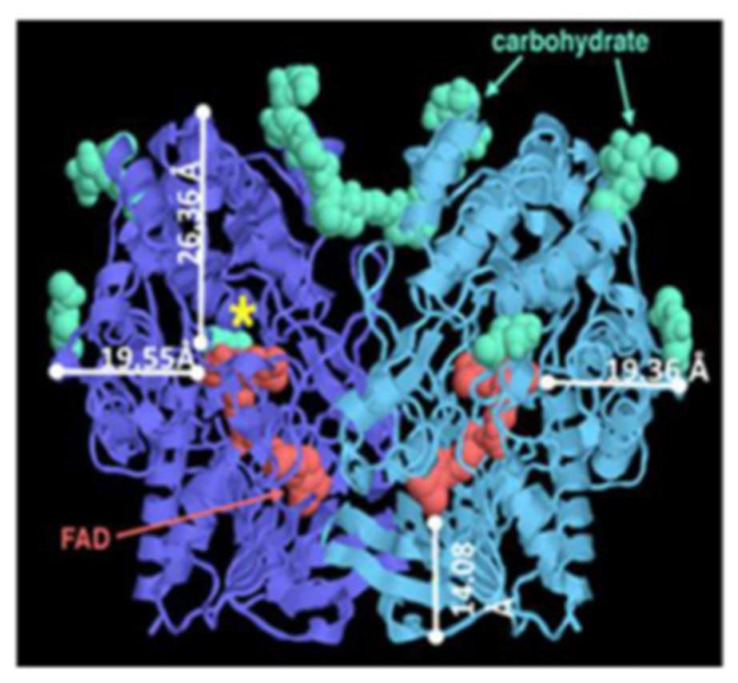
Gox structure with FAD co-factors bound deep inside the enzyme red color. Reprinted with permission from ref. [91]; Copyright 2012 Elsevier.

Glucose oxidase (GOx) and glucose dehydrogenase (GDH) are two types of enzymes that are frequently utilized in glucose biosensors as the catalyst for the glucose redox reaction. These enzymes differ in terms of redox potential, cofactors, and D-glucose selectivity [86]. GOx enzyme is widely explored due to its easy handling and high substrate specificity in a glucose environment [87]. The fungus *Aspergillus niger* is frequently used to manufacture GOx enzyme. GOx enzyme is a homodimer made of two identical subunits and one non-covalently bound flavin adenine dinucleotide (FAD) co-enzyme tightly bound in the active site of the enzyme, as shown in Figure 4. FAD is in funnel-shaped active sites with an opening of 100 Å [48]. FAD plays a role as a redox cofactor (coenzyme), which uses oxygen as the external electron acceptor, releases hydrogen peroxide (H_2_O_2_), and acts as an electron carrier during catalysis [88]. In glucose biosensor application, the GOx enzyme offers the advantages of cost effectiveness and high stability, but is dependent on the oxygen content in the electrolyte solution [89].

Instead of using oxygen as the electron acceptor, the GDH enzyme transfers electrons to a variety of organic and synthetic electron acceptors. GDH is a monomer that consists of two domains: a central nucleotide as the binding domain, and flanked by the catalytic domain. The GDH enzyme is categorized based on the co-factor, which is mainly classified into three cofactors: pyrroloquinoline quinone (PQQ), nicotinamide adenine dinucleotide (NAD), and FAD [90]. However, the GDH enzyme’s limitation is dependent on the type of co-factor used. FAD-GDH is expensive and involves a lengthy preparation process, and PQQ-GDH has poor selectivity due to susceptible interference from a variety of saccharides. NAD-GDH exhibits excellent selectivity and stability, but is limited in finding a match with mediator properties.

#### Enzyme Immobilization Technique

**Figure 5:**
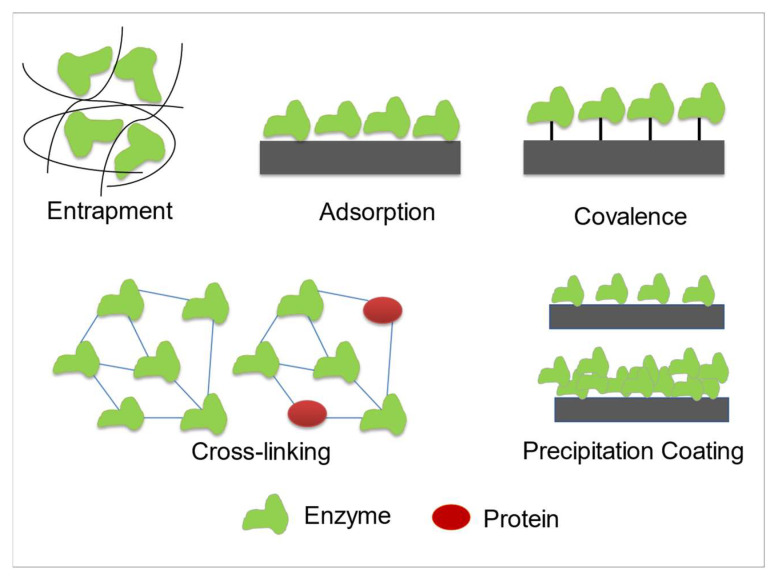
Schematic of the main methods for enzyme immobilization.

In enzymatic glucose biosensors, glucose oxidase and glucose dehydrogenase are the common enzymes employed to develop glucose biosensors. The performance of glucose biosensors is very much influenced by three main factors, which are morphology, structure, and enzyme immobilization technique. Appropriate GOx enzyme immobilization onto the nanomaterial-modified electrode surface is essential to ensure a stable and efficient enzymatic glucose biosensor. Enzyme immobilization can be defined as the physical confinement or localization of enzymes in a certain region of space while maintaining their catalytic activities and being able to be used repeatedly. Good immobilization of the enzyme should be located close to the surface of the modified electrode, maintain bioactivity, prevent enzyme leakage, and prolong the lifetime of the working electrode [92,93]. The five most commonly used methods for GOx enzyme immobilization are adsorption, covalent, cross-linking, electrodeposition, and polymer entrapment, as shown schematically in Figure 5 [92,94].

Enzyme immobilization via adsorption is a very simple and widely used method with little or no distortion in the enzyme structure. In this approach, the non-covalent linkage between the enzyme and surface of the modified electrode can occur through weak non-specific forces (hydrophobic interaction, hydrogen bond, and Van der Waals), ionic bonding (salt linkages), or electrostatic absorption [94]. Thus, the enzyme immobilizes in random orientation on the nanomaterial-modified electrode surface. Several studies reported that enzyme immobilization via adsorption only requires soaking or drop-casting of the enzyme solution onto the surface of a nanomaterial-modified electrode, and it is incubated overnight or 24 h to allow physical adsorption to occur [95,96,97,98]. Although this method ensures a natural conformation of enzyme, this method suffers major disadvantages such as enzyme leakage and desorption of the enzyme with changes in the temperature, pH, and ionic strength of the analyte solution [94].

Enzyme immobilization by covalent binding involves the formation of a covalent bond for sharing of electron pairs between one or more of enzyme’s functional groups, either with the surface of electrode or onto a thin membrane attached on the electrode [99]. The covalent binding of enzyme with the surface of the nanomaterial-modified electrode requires activation using multifunctional reagents such as glutaraldehyde and carbodiimide (EDC-NHS). Through covalent bonding, the orientation of GOx attachment can be controlled through chemical bonding. The advantages of immobilization by covalent bonding are minimum leaching of the enzyme, as it is tightly bound with the nanomaterial-modified electrode compared with adsorption bonding and ultimately increases the stability of the glucose biosensor [94,99]. Additionally, covalent bonding can introduce the path for electron transfer between the deeply buried active FAD-redox center of the enzyme and the nanomaterials modifying the surface electrode. However, immobilization via covalent binding is more expensive due to its complexity as reactions need to occur at low temperatures, and the functional group present on the nanomaterial-modified electrode needs to be activated prior to enzyme immobilization [100]. Another limitation of enzymatic covalent binding is that the enzyme is regenerable and enzymatic activity decays.

Cross-linking enzyme immobilization can be performed by forming a cross-linking bridge between enzyme via bi- or multifunctional reagents on a nanomaterial-modified electrode. Normally, cross-linking molecules contain two or more reactive ends for chemically bridging to specific functional groups of an enzyme. The commonly used cross-linking reagents are glutaraldehyde, glyoxal, and hexamethylnediamine [99]. Cross-linking between two functional groups of a single enzyme is known as intra-molecular crosslinking that stabilizes an enzyme’s internal structure, whereas inter-molecular crosslinking involves bridging groups of two different units of enzymes to stabilize an enzyme–enzyme interaction [92]. However, the limitation of cross-linking enzyme immobilization is the enzyme activity decay due to the chemical modification of the active site enzyme and distortion of enzyme structure during cross-linking interaction.

Previously, Jung and Lim [101] presented the effect of different coupling agents from aminosilane (AS) group, such as (3-aminopropyl)triethoxysilane (APTMS), 3-aminoporpyltriethoxysilane (APTES), and 3-aminopropylmethyldiethoxysilane (APS), forming covalent binding with immobilized GOx enzyme. They reported that APS gave the highest sensitivity of 17.72 μAcm^−2^ mM^−1^ compared with other samples because of high GOx enzyme loading in APS coupling agents and low electron transfer resistance for efficient electrocatalytic activity with glucose. Shukla et al. [102] studied the glucose sensor performance via two different immobilization methods (physical adsorption and cross-linking). They found that the sensitivity and linear range of sensors improve when the cross-linking method is used, which is due to an increase in enzyme loading onto the ZnO nanorod (NR) surface and less enzyme leaching compared with the physical adsorption immobilization. Lipińska et al. [103] compared three GOx enzyme immobilization strategies on the Au-Ti heterostructure-modified electrode: adsorption, covalent, and cross-linking. They found that cross-linking of the GOx enzyme with Au-Ti hydrostructure produces a glucose biosensor with excellent electrochemical performance for invasive glucose detection in the linear detection range of 0.05–3.05 mM and LOD of 7.61 µM. High kinetic interactions with glucose occur due to high GOx enzyme loading through the cross-linking reaction. Additionally, the bovine serum albumin moieties help preserve the GOx enzyme activity on the Au-Ti heterostructure-modified electrode.

Kowalewska and Jakubow [104] also reported the impact of immobilization on the conformation of GOx bioactivity and electrochemical performance. In general, GOx enzyme consists of a number of amino acids, which are arranged into helices or sheets. Therefore, Fourier-transform infrared spectroscopy (FTIR) was used to study the conformational changes in the structure of GOx after cross-linking, and covalent binding immobilization was applied. FTIR analysis showed that pure GOx consists of ~24% α-helices, 13% β-sheets, ~30% β-turns, 2% antiparallel β-sheets, and ~32% random coils. After covalent binding immobilization, the percentage of α-helices decreases, whereas the percentage of antiparallel β-sheets increases. This indicated a definite change in the tertiary structure of GOx molecule, which suggested a denaturation of immobilized GOx molecules by covalent binding. As a result, low sensitivity, small linear range, and slow heterogeneous electron transfer rate of the electrode were observed compared with the sensor immobilized via the cross-linking method.

**Figure 6:**
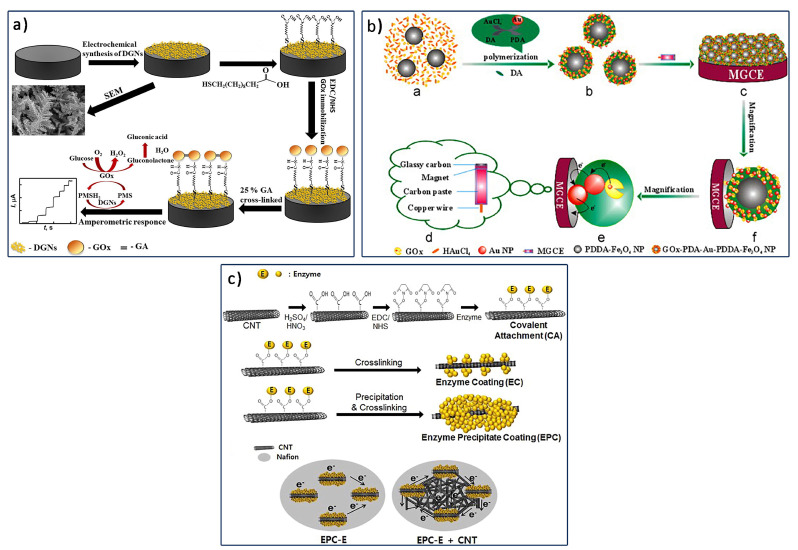
Schematic of fabrication of (**a**) GOx−Au nanostructure/graphite electrode using additional crosslinking reaction after GOx covalently binds with self-assembled monolayer. Reprinted with permission from ref. [105]; Copyright 2022 MDPI. (**b**) Polymerized GOx−AuNP−polydopamine−IONP−modified GCE electrode for glucose biosensor application. Reprinted with permission from ref. [31]; Copyright 2013 Elsevier. (**c**) GOx/CNT/GCE−modified electrode via EPC approach of GOx immobilization. Reprinted with permission from ref. [107]; Copyright 2015 Elsevier.

Recently, a graphite rod modified with dendritic Au nanostructure, GOx enzyme, and phenazine methosulfate as the soluble redox mediator was developed for use as an electrochemical glucose biosensor [105]. In their work, three GOx enzyme immobilization approaches were compared: GOx cross-linking using glutaraldehyde, GOx covalent immobilization using self-assembled monolayer, and additional GOx cross-linking on the covalently bind self-assembled monolayer, as shown schematically in Figure 6a. Among all enzyme immobilization approaches studied, the additional cross-linking approach after covalent GOx enzyme binding with the self-assembled monolayer showed 1.41 times higher peak current generated during the enzymatic reaction compared with the two other methods. The multilayer enzyme structure on the Au nanostructure provided a large electrochemical active surface area and excellent electron transfer.

Another interesting method to immobilize enzyme is via electropolymerization, where an enzyme is immobilized in 3D matrices, such as an electropolymerized film, an amphiphilic network, a photopolymer, a silica gel, a polysaccharide, or a carbon paste. Using this method, enzymes, mediators, and additives can be immobilized simultaneously on the same sensing layer, so any modification to the biological molecules is not required. This method is simple and guarantees that the enzyme is well preserved during the immobilization process. The drawback of this method lies on possible leaching of the biocomponent and limitation in the performance of the glucose biosensor due to the presence of a diffusion barrier [106].

Peng et al. [31] modified the GCE electrode using a physically entrapped GOx enzyme in a polymerized nanocomposite of GOx-AuNP-polydopamine-IONPs, as illustrated schematically in Figure 6b. The modified electrode not only has the magnetism of IONPs, which allows them to be easily manipulated by an external magnetic field, but it also has polydopamine’s excellent biocompatibility to maintain the native structure of GOx and AuNPs’ good conductivity, which can facilitate direct electrochemistry of GOx in the biofilm. Thus, the presence of GOx-AuNP-polydopamine-IONP/GCE displays a good linear amperometric response to glucose concentrations ranging from 0.02 mM to 1.875 mM.

Another interesting approach in GOx enzyme immobilization is the enzyme precipitation coating (EPC). The EPC approach involves three basic steps of covalent binding of GOx enzyme: GOx enzyme precipitation via addition of salts, organic solvents of polymeric materials, and cross-linking of GOx enzyme with bifunctional reagents. Previously, Kim et al. [108] reported on the high-stability GOx enzyme through the EPC approach on the electrospun polymer nanofibers and CNTs for glucose detection. They reported [107] EPC-GOx-CNT-modified GCE. The fabrication procedure is schematically shown in Figure 6c. Higher enzyme loading due to EPC improved the electron transfer rate of the modified electrode for glucose detection. Additionally, covalent bonding improved the enzyme stability by preserving denaturation and enzyme leakage. Recently, Bi et al. [109] reported on Au nanodendrite and GOx immobilized via EPC for the modification of flexible carbon fiber cloth. The nanodendrite structure provided a large surface area for GOx loading through EPC. With high GOx loading, wide-linearity and high-stability glucose biosensors were developed.

### 3.3. Electrode Materials

In electrochemical glucose biosensors, the sensor performance is controlled by the interaction that occurs between the working electrode and the electrolyte solution interface. Therefore, types of working electrode and their surface structure are important aspects that control the efficiency of the electrochemical performance of glucose biosensors. Electrochemical glucose biosensors that have excellent sensitivity, good reproducibility, and low-cost working electrodes are always in demand for glucose biosensors. Previous research reported the modification of electrodes for glucose biosensors using solid carbon electrodes (e.g., magnetic glass carbon electrode and carbon paste electrode) [95,110] and solid noble metal electrodes (e.g., Pt and Au) [111,112]. The limitation of using solid bulk electrodes is the difficulty to transfer the development process into the disposable electrode to suit the application for home blood glucose monitoring. A disposable electrode offers advantages for fast screening and on-site monitoring because of its low cost, high sample throughput, and easy integration into mass production processes [113]. A disposable working electrode can be categorized into carbon-based, glass-based, or flexible-based electrodes; it is commonly fabricated using screen printing, casting, deposition, and sputtering techniques. Table 2 lists the types of disposable electrode, advantages, disadvantages, and examples of each type of disposable electrode.

The disposable electrode is a suitable platform for glucose biosensors due to its miniaturization, portability, and economic benefits for home blood glucose monitoring. Disposable electrodes are modified with various types of nanomaterials to improve the electrochemical performance in glucose detection. The surface modification techniques employed are drop casting, dip coating, electrodeposition, and the direct growth technique. Among all disposable electrodes, SPCE offers simplicity because the whole electrochemical electrode system consists of reference, working, and counter electrodes integrated on the same substrate. Compared with SPCE, the ITO and FTO electrodes possess high-temperature stability, thereby allowing surface modification with nanomaterials via the direct growth technique, which commonly requires annealing at high temperatures. Currently, the disposable electrode with flexible characteristics is widely explored as the working electrode for glucose biosensors. The reason is that the ability to bend and stretch make it suitable to be applied for wearable and continuous glucose biosensors.

### 3.4. Type of Electrolyte and pH of Electrolyte

In enzymatic glucose biosensors, phosphate buffered saline (PBS) is commonly employed as an electrolyte because the ion concentration and osmolality of PBS closely mimic the human body. The pH of PBS is the most important factor that controls the electrochemical signal of a glucose biosensor’s performance. The structure and shape of an enzyme vary depending on the pH of the electrolyte. The changes to the structure affect the activity of the enzyme. The pH of the electrolyte can change the ionization state of the amino group of enzymes, which is commonly a reversible process [123].

The redox behavior of the GOx enzyme is influenced by the proton (H^+^) and hydroxide (OH^−^) concentration present in the electrolyte. At acidic conditions (<pH 4), GOx activity decreases due to denaturation. At alkaline conditions (>pH 8), GOx activity decreases because of low proton concentration. The GOx enzyme exhibits optimum redox behavior at neutral pH conditions (pH 6.8–7.4).

As for the non-enzymatic glucose biosensor, the common condition of electrolytes is alkaline with pH 8–10. NaOH electrolyte with a concentration of 0.1–0.5 M is usually employed for the glucose oxidation reaction. Alkaline condition is important because the OH^−^ of alkaline electrolyte promotes the catalytic reaction of β-D-glucose. Many studies have investigated non-enzymatic glucose biosensors under neutral (pH 7–7.4) electrolyte conditions. The pH conditions of human body fluid (serum, tears, and sweat) used as a sample in glucose biosensor are in neutral pH. Thus, a working electrode with bimetallic compound and porous nanomaterials to accelerate electron transfer and enhance the catalytic reaction during glucose detection was developed.

Recently, Goodnight et al. [124] reported on electrochemically modified Cu-Ni electrode for a non-enzymatic glucose biosensor tested in neutral PBS electrolyte solution. The bimetallic modified electrode was prepared by sequential electrodeposition of the Cu and Ni nanostructure using an electron beam technique, followed by an annealing process. Recently, Wang et al. [34] reported the modification of non-enzymatic Au electrode with Pt shell on Pd nanocubes (NCs) as the catalyst for the glucose oxidation reaction under neutral condition using PBS electrolyte. The application of bimetallic compound of Pt-Pd and Cu-Ni caused high activity and improved durability in hydrogen evolution reactions. As for porous nanomaterial-modified electrodes, Lee et al. [125] developed SPCE modified with Pt nanoparticles with porous structure for the non-enzymatic glucose biosensor operated in neutral (pH 7.4) PBS electrolyte condition. The nanoporous structure can increase the electrochemical active sites for electron transfer during the glucose oxidation reaction.

Strakosas et al. [126] invented bioelectronic pH control integrated with the non-enzymatic glucose biosensor operated in neutral (pH 7) conditions. The bioelectronic control of the pH condition was achieved by stimulating a localized and reversible pH change, which absorbed H^+^ from neutral fluid and increased the pH, thereby enabling glucose sensing in neutral biological fluid.

## 4. Recent Development of Nanomaterial-Modified Electrode for Enzymatic Glucose Biosensor

In enzymatic glucose biosensors, the glucose detection mechanism is based on immobilized GOx, which catalyzes the oxidation of glucose and conversion into gluconic acid. In the first-generation glucose sensor, the glucose detection mechanism is dependent on H_2_O_2_ generation or decreases in O_2_ concentration. In the second-generation glucose sensor, the glucose detection mechanism is dependent on the redox reaction of the mediator. In the third-generation glucose sensor, the glucose detection mechanism is based on direct electron transfer between the enzyme and electrode. In a conventional electrochemical system, unmodified working electrodes of enzymatic glucose biosensors have the limitation of embedded FAD redox active center of GOx enzyme that blocks electron transfer between the enzymes and electrode, resulting in the reduction of electrochemical performance.

Recent developments of glucose biosensors involve modifying the working electrode with nanomaterials, such as noble metal, metal oxide, and carbon-based materials. All nanomaterials can be synthesized into various types of nanostructures, such as nanoparticles, nanotubes, nanorods (NRs), and hierarchical nanostructures, which further enhance the performance and stability of the fabricated glucose biosensor. The incorporation of nanomaterials or nanostructured materials as matrix/transducing element in enzymatic glucose biosensor provides a series of advantages such as large surface area for enzyme immobilization and high catalytic efficiency, which then enhance the electron transfer behavior between enzymes and the electrode.

Several factors can influence the analytical and lifetime stability performance of enzymatic glucose biosensors, such as electrode materials, physical and chemical properties of the nanomaterials, functional group or polymer coating, structure and morphology of the electrode, nanomaterial modification technique, and enzyme immobilization technique. Many immobilization methods have been proposed to increase the enzyme loading and stability including covalent attachment, cross-linking, physical entrapment, and adsorption. For commercialization in glucose clinical analysis, it is vital for the fabricated enzymatic nanomaterials modified electrode able to meet the necessary standards lifetime of commercial glucose biosensors by retaining 85% enzyme activity within 35–50 days.

### 4.1. Metal-Based Enzymatic Glucose Biosensor

Various types of noble metal nanomaterials and alloys were employed for the modification of enzymatic glucose biosensors, such as Au, Pt, Pd, Cu, and Ag. Noble metal nanoparticle structures offer unique electronic and electrocatalytic properties, which ensure electrochemical reversibility for the redox reaction. Among all noble nanomaterials, Au and Pt nanomaterials are commonly employed in the modification of electrodes for glucose biosensors. In general, Au and Pt nanomaterials are chemically stable, optically sensitive, biocompatible, and catalytically active in the oxidation reaction of glucose, making them interesting candidates in the modification of working electrodes for electrochemical glucose biosensors. Several shapes of Au and Pt nanostructure materials have been investigated; these shapes include nanoparticles, rod-shaped nanoparticles, nanoporous films, and nanowires.

The modification of Au and Pt nanomaterials is commonly conducted via absorption (drop-cast or immersion), self-assembly to the active functional group on the electrode surface, and electrodeposition. Among those techniques, electrodeposition is the preferred technique for electrode modification. The electrodeposition technique is a simple and fast method for the immobilization of nanomaterials on various working electrode shapes. The electrodeposition process offers uniform nanomaterial distribution due to simple process control. The Au and Pt nanomaterials commonly combine with other metal, metal oxide, carbon-based, and polymeric materials, forming hybrid/composite nanomaterials for modification of the working electrode. This phenomenon further enhances the electrocatalytic properties and facilitates electron transfer between GOx and electrode surface of the modified electrode during glucose detection. Table 3 lists a summary of enzymatic glucose biosensors based on metal nanomaterials and nanohybrid/nanocomposite.

**Figure 7:**
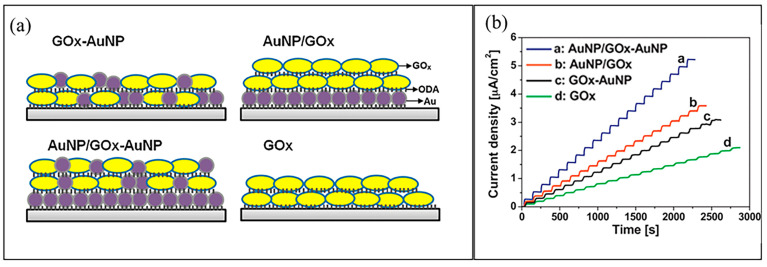
Four structural designs of GOx and AuNP composite film-modified ODA-Pt electrodes: (**a**) schematic and (**b**) amperometric response. Reprinted with permission from ref. [127]; Copyright 2016 Elsevier.

Wang et al. [127] studied the effect of GOx-AuNP composite structural design developed using a new technique of Langmuir–Blodgett deposition for the modification of Pt electrode in amperometric glucose detection. The Langmuir–Blodgett deposition technique is the enzyme immobilization technique that utilizes the adsorption of GOx enzyme molecules and AuNPs on the electrode with the ability to control the structure and film thickness of the composite. The Pt electrode was functionalized with an octadecylamine (ODA) template monolayer before the adsorption of the GOx-AuNP composite layer. Four structural designs of GOx-AuNP composite-modified ODA-Pt electrode were studied; the first condition consisted of two layers of mixed GOx-AuNP monolayer (GOx-AuNPs); the second condition consisted of one layer of close-packed AuNPs and two layers of GOx enzyme (AuNP-GOx); the third condition consisted of one layer of close-packed AuNPs and two layers of mixed GOx-AuNPs (AuNP/GOx-AuNP); and the fourth condition consisted of two layers of GOx without AuNP layer, as shown schematically in Figure 7a. Figure 7b shows the amperometry response for the four structural designs of the GOx-AuNP composite electrodes with linear detection in the glucose concentration range of 0.05–5 mM. The sensitivity of the four structural designs of GOx-AuNP composite electrodes is ranked as AuNP/GOx-AuNP (third condition) > AuNP/GOx (first condition) > GOx-AuNPs (second condition) > GOx (fourth condition). The modification of AuNPs, regardless of the close-packed structure or a mixed layer, shows improvement in the sensitivity of glucose detection due to excellent electrical and electrocatalytic performance of the AuNPs. The structural orientation of the GOx and AuNP composite showed that the composite film arranged layer-by-layer presented better electrochemical performance compared with the simultaneous assembly of the composite film. The glucose detection mechanism of the modified electrode in this work is based on the electrochemical reduction of O_2_ and electrochemical oxidation of the product H_2_O_2_.

In enzymatic glucose biosensors, electrodes are commonly modified with nanocomposite or nanohybrid materials instead of single-type nanomaterials. The combination of individual properties can achieve better electrochemical performance of the modified electrode for glucose detection than single-type nanomaterials. Technically, there are differences between nanocomposite and nanohybrid materials. Nanocomposites are multiphase materials, which combine one or more phases of nanomaterials with distinct physical or chemical properties. In general, nanocomposites incorporate nanomaterials into a second phase of materials known as a matrix. Nanocomposites can be classified based on the types of matrix materials, such as ceramic, metal, and polymer. In nanocomposites, new properties that are not present in individual materials can be created [128]. Nanohybrid is the connection between one or more organic and inorganic nanomaterials by covalent or non-covalent binding, and it can develop new properties through the synergism of individual properties. Nanohybrids are commonly fabricated through polymerization, sol-gel, and organic functionalization [129].

Applications of polymeric materials in the modification of glucose biosensor electrodes have attracted extensive attention. The conducting polymer, such as polyacetylene, polypyrrole (PPy) [122,130], polyaniline (PANI) [131,132], poly(3,4-ethylenedioxythiophene) (PEDOT) [68], and polyphenylene, has been utilized in the modification of enzymatic glucose biosensors. Conducting polymers have excellent electrical conductivity, functional group for the immobilization of GOx enzyme, and chemical stability. Additionally, conjugated polymers were obtained by electropolymerization (P) processes; 10,13-bis (4-hexylthiophene-2-yl) dipyridol [3,2-a: 2′,3′-c] phenazine (HTPP) monomer forming P(HTPP), poly (ditieno (3,2-b: 2′,3′-d) pyrrole) (DTP) monomer forming P(DTP), and 3-(5,8-bis (2,3-dihydrothieno [3,4-b][1,4]dioxin-5-yl)-3-(9-hexyl-9H-carbazole-3-yl)quinoxalin-2-yl)-9-hexyl9H-carbazole (HCQE) monomer forming PHCQE have been applied in the modification of glucose biosensors. The conjugated polymers have advantages of adjustable band gap, amine or carboxylic groups that are suitable as GOx immobilizer, and good electrical conductivity. Despite the exceptional properties of the conducting and conjugated polymer, they do not have catalytic properties. Thus, to be applied in the modification of glucose biosensors, polymeric materials are combined with metal or metal oxide materials [133].

Zhang et al. [134] developed polylactic acid (PLA)-Au microneedle modified with overoxidized PPy (OPPy), AuNPs, GOx enzyme, and Nafion layer for invasive glucose monitoring. The Nafion/GOx/AuNPs/OPPy/Au-microneedle-modified electrode showed linearity for glucose detection from 0 mM to 2.6 mM with a good sensitivity of 8.09 μA/mM and low LOD of 40 μM. In their work, PLA played a role in improving the mechanical stability of microneedles during skin injection. Additionally, the overoxidized PPy provided a suitable surface area for the deposition of AuNPs and binding of GOx enzyme. The cross-linking of GOx enzyme using glutaraldehyde and the presence of Nafion layer produced good electrocatalytic properties and mechanical stability to the Nafion/GOx/AuNP/OPPy/Au-microneedle-modified electrodes during glucose detection. In this work, the glucose detection mechanism was based on the electrochemical reduction of O_2_ and electrochemical oxidation of the product H_2_O_2_.

Kim et al. [135] developed Au microneedle arrays modified with terthiophene carboxylic acid (TCA) conductive polymer as a mediator and enzyme immobilizer. The -NH_2_ group of the GOx enzyme was covalently bound to the -COOH group of TCA/Au microneedles via amide bond. Excellent sensitivity of 0.22 μA/mM^−1^ cm^−2^ was achieved for wide linear glucose detection of 0.05–20.0 mM, and selective glucose biosensors were developed. The covalent immobilization of the GOx enzyme of the modified electrode ensured excellent catalytic properties of the GOx/TCA/Au microneedle-modified electrode. In their work, the glucose detection mechanism was based on the electrochemical reduction of O_2_ and electrochemical oxidation of the produced H_2_O_2_.

The enzymatic glucose biosensor based on the conjugated polymer-AuNP bio composite was developed by Tan and Baycan [136]. The graphite pencil electrode (GPE) was modified with electropolymerization of the monomer 3-(5,8-bis (2,3-dihydrothieno [3,4-b][1,4]dioxin-5-yl)-3-(9-hexyl-9H-carbazole-3-yl)quinoxalin-2-yl)-9-hexyl9H-carbazole (HCQE), AuNPs, and GOx enzyme. GOx was cross-linked with glutaraldehyde on the PHCQE/AuNP/GPE-modified electrode using the immersion technique. The GOx/AuNP/PHCQE/GPE-modified electrode showed sensitivity of 0.13 μA/mM^−1^ for the linear detection of 0.75 and 3.125 mM and LOD of 17 μM. The conjugated PHCQE polymer with AuNPs provided an ideal surface area for GOx immobilization. Good GOx adherence on the AuNP/PHCQE/GPE-modified electrode was achieved due to strong π–π* interaction and the presence of hydrophobic alkyl chains in the backbone of the PHCQE polymer structure. The glucose detection mechanism in this work was based on the electrochemical reduction of O_2_ and electrochemical oxidation of the produced H_2_O_2_.

To further enhance the electrocatalytic properties of the enzymatic glucose biosensor, the combination of conducting and conjugated polymeric materials with Pt nanomaterials has been explored. Zhai et al. [133] reported on the modification of Pt electrode with PANI hydrogel and PtNPs for high loading of the GOx enzyme. The 3D porous structure of PANI hydrogel allowed high density of PtNP attachment, favoring high loading of GOx enzyme. The GOx/PtNP-PANI/Pt-modified electrode showed high sensitivity of 96.1 μAmM^−1^ cm^−2^ with linearity glucose concentration of 0.01–8.0 mM and very low LOD of 0.7 μM. The synergistic advantage of PANI hydrogel together with PtNPs improved the electrocatalytic performance of the GOx/PtNP-PANI/Pt-modified electrode during glucose detection. In another work [137], the combination of o-phenylenediamine (oPD) and polyvinylferrocenium perchlorate (PVF-ClO_4_^−^) polymeric materials together with PtNPs and immobilization of GOx enzyme was developed to modify Pt electrode, which was denoted as Gox-PoPD/PtNPs/PVF/Pt. The fabrication of the modified electrode started with electroprecipitation of PVF-ClO4^−^, followed by electrodeposition of PtNPs. Finally, GOx enzyme was electropolymerized with oPD. In this work, PVF-ClO4^−^ functioning as an excellent mediator with good stability was combined with oPD to further improve the electrical conductivity, surface area for enzyme immobilization, and mechanical properties of the Gox-PoPD/PtNP/PVF/Pt-modified electrode.

Recently, activated SPCE was modified with GOx enzyme, PtNPs, and poly(Azure A) (PAA) for a glucose biosensor [138]. PAA was electropolymerized on the SPCE, followed by electrodeposition of PtNPs. Finally, the GOx enzyme was drop-casted on the PtNP/PAA/SPCE-modified electrode. The GOx/PtNP/PAA/SPCE-modified electrode exhibited high sensitivity of 42.7 μA mM^−1^ cm^−2^ for linearity of 20 μM–2.3 mM and LOD of 7.6 μM for amperometry glucose detection at low potential of 0.2 V.

Most commercially available electrochemical enzyme glucose strips use an artificial electron mediator to transfer electrons generated from the active sites of the GOx enzyme to the electrode. Mediators were used to lower the redox potential, which avoids oxidation of interfering species. However, as a result of their small and diffusive molecules, mediators may leak and react with interfering species, thereby affecting the efficiency and accuracy of glucose analysis [59]. Eventually, this decreases the operational lifetime and efficiency of the modified electrode. To overcome this problem, many researchers have focused on utilizing nanomaterials with different structures for the modification of working electrodes, which minimize mediator leaching and increase the electron transfer rate between GOx enzymes and electrode during glucose detection [33].

German et al. [139] developed an enzymatic glucose biosensor based on the modification of graphite rod electrode with GOx enzyme, AuNPs, and PPy conducting polymer. Phenazine methosulfate (PMS) was applied as a redox mediator in this work. Initially, 13 nm AuNPs synthesized through chemical reduction were electrochemically deposited on the graphite rod electrode. The GOx enzyme was immobilized on the AuNPs/graphite rod through cross-linking using glutaraldehyde. The Ppy conducting polymer was electropolymerized to cover the GOx/AuNP/graphite rod-modified electrode. In this work, the effect of PPy electropolymerization time (21, 38, and 104 h) on the electrochemical glucose biosensor performance was studied. The glucose detection mechanism was based on the redox reaction of PMS/PMSH_2_, which transferred the electrons via two approaches, either directly to the graphite rod electrode or through AuNPs. The electrochemical performance of the PPy/GOx/AuNP/graphite rod-modified electrode showed that prolonged electropolymerization time of PPy from 21 h to 104 h increased the PPy film thickness, which widened the linear glucose concentration detection range of glucose. This finding was due to the fact that the thicker PPy film reduced the diffusion of glucose and PMS as mediator. The applicability of the modified electrode was tested in human serum samples with good recovery (97–99%).

**Figure 8:**
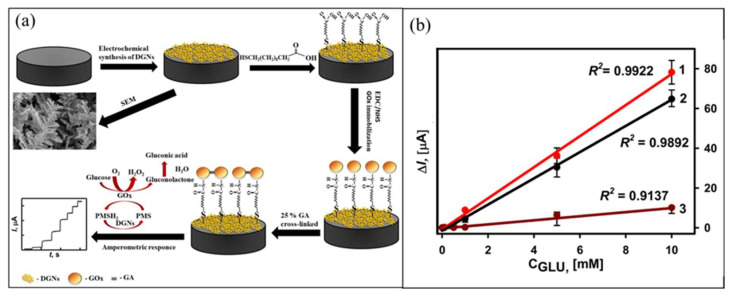
(**a**) Schematic of GOx immobilization by the third method of GA-GOx-SAM/x Au-nanostructure/graphite rod. (**b**) The calibration plot of amperometric response of GA-GOx/dendritic Au-nanostructure/graphite rod (curve 1, third method), GOx-SAM/dendritic Au-nanostructure/graphite rod (curve 2, first method), and GA-GOx-SAM/dendritic Au-nanostructure/graphite rod (curve 3, second method)-modified electrode. Reprinted with permission from ref. [105]; Copyright 2022 MDPI.

Sakalauskiene et al. also utilized PMS as redox mediator [105]. In their work, the comparison of GOx immobilization technique on the dendritic Au nanostructured-modified graphite rod electrode for glucose biosensor was examined. Three GOx immobilization techniques were employed: the first method was by adsorption and cross-linking with glutaraldehyde (GA) (GA-GOx/dendritic Au nanostructure/graphite rod), the second method was by covalent immobilization and modification with 11-mercaptoundecanoic acid self-assembled monolayer (SAM) (GOx-SAM/dendritic Au nanostructure/graphite rod), and the third method was by covalent immobilization on SAM with additional cross-linking with GA (GA-GOx-SAM/dendritic Au nanostructure/graphite rod). The team reported that GA significantly improved the stability of the enzyme layer. As observed in the calibration plot of the amperometric response for the modified electrode in Figure 8b, the GA-GOx-SAM/dendritic Au nanostructure/graphite rod immobilized with the third method showed the highest electrochemical response, followed by GA-GOx/dendritic Au nanostructure/graphite rod immobilized with the second method, and the lowest was the GOx-SAM/dendritic Au nanostructure/graphite rod immobilized by the second method. Combining covalent and cross-linking to immobilize GOx in the third method greatly increased the sensitivity, specificity, and stability of the modified electrode for glucose detection. The strong binding caused by the covalent and cross-linking of the GOx enzyme to the high surface area of dendritic Au nanostructure/graphite rod minimized the loss of enzyme and improved the electrocatalytic performance of the modified electrode for glucose detection. The GA-GOx-SAM/dendritic Au nanostructure/graphite rod showed linear detection in the range of 1–10 mM and LOD of 19 µM.

**Figure 9:**
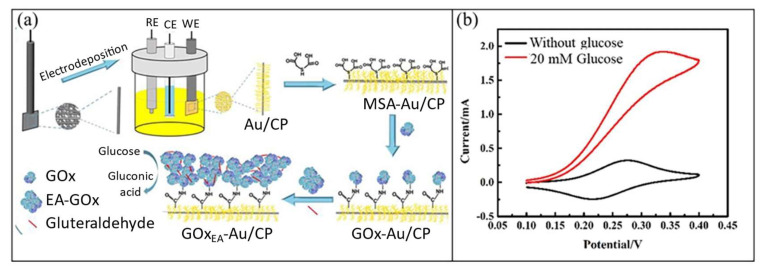
(**a**) Schematic of the fabrication process of the GOx/3D Au coral/carbon paper−modified electrode, (**b**) CV of the GOx/3D Au coral/carbon paper−modified electrode in PBS solution (pH 7) containing ferrocene as redox mediator without glucose and with 20 mM glucose. Reprinted with permission from ref. [140]; Copyright 2020 Elsevier.

Another interesting approach of preparing high loading of the GOx enzyme on the matrix nanomaterials with high surface area is by using 3D nanostructure materials, such as dendrite nanostructure, porous nanostructure, foam nanostructure, and coral nanostructure. Yan et al. [140] reported the modification of porous carbon paper with 3D Au coral nanostructure and GOx enzyme. The schematic of the formation of the GOx/3D Au coral/carbon paper-modified electrode is shown in Figure 9a. The 3D Au coral nanostructure was prepared through electrodeposition on the carbon paper aided by H_2_ evolution. The 3D Au coral/carbon paper-modified electrode was functionalized by immersion in mercaptosuccinic acid (MSA) solution. The GOx enzyme was immobilized using a combination of techniques of covalent attachment using EDC/NHS and cross-linking enzyme aggregates using glutaraldehyde. Ferrocene was used as the redox mediator during glucose detection.

The mechanism of glucose detection for the GOx/3D Au coral/carbon paper-modified electrode was as in the second-generation glucose biosensor, which uses ferrocene as mediator. Figure 9b shows the cyclic voltammetry (CV) of the GOx/3D Au coral/carbon paper-modified electrode in PBS solution (pH 7) containing ferrocene as the redox mediator without glucose and with 20 mM glucose. Well-defined redox peaks were observed without glucose, which was caused by the oxidation and reduction of the ferrocene mediator. When 20 mM glucose was added, the oxidation peak sharply increased, whereas the reduction peak sharply reduced. This result was observed due to the rapid electron transfer between reduced GOx (GOx-FADH_2_) and oxidized forms of ferrocene (Fc_ox_). The GOx/3D Au coral/carbon paper-modified electrode showed excellent performance in glucose detection with wide linearity of 0.002–21.97 mM, high sensitivity of 96.27 μA mM^−1^ cm^−2^, and low LOD of 0.6 µM. The excellent electrocatalytic performance of the GOx/3D Au coral/carbon paper-modified electrode in glucose detection was caused by the large active surface area of the 3D Au coral nanostructure, which allowed high loading of GOx enzyme. Additionally, the covalent adherence and cross-linking aggregation of enzyme ensured strong binding of the GOx enzyme on the 3D Au coral/carbon paper-modified electrode. The modified electrode also showed good feasibility and reliability for glucose biosensor application in human serum samples.

Carbon-based materials such as CNTs and graphene are normally combined with metal, metal oxide, or polymeric materials, forming nanocomposite and nanohybrid materials for the modification of glucose biosensor electrodes. In general, carbon-based materials have high electrical conductivity, low background current, wide potential window, excellent electron transfer capabilities, and large surface area [141]. Carbon-based materials are characterized by a variety of electrochemical characteristics depending on their diverse morphology: 1D of CNTs, 2D of graphene and graphitic carbons, and zero-dimensional fullerenes and carbon quantum dots. However, CNTs and graphene are hydrophobic, so they require functionalization or hybridization with some inorganic materials.

Utilizing noble metals in the modification of electrodes requires high catalytic potential, which may cause oxidation of interference substances such as ascorbic acid, uric acid, sucrose, and galactose. This weakness can be overcome with graphene. Graphene can connect the FAD active center of the GOx enzymes with the electrode surface, thereby inducing direct electron transfer [142]. Graphene also has the capability to reduce the working potential during electrochemical glucose detection.

To confirm that direct electron transfer between FAD active center of the GOx enzyme with electrode surface was achieved, the determination of glucose must be conducted in the absence of O_2_. If the analysis of glucose detection is conducted under the presence of O_2_, it should be classified as a first-generation glucose biosensor rather than as a third-generation biosensor. Rafighi et al. [111] developed a direct electron transfer-modified electrode for glucose biosensor based on the modification of Au electrode by immobilization of GOx enzyme on nanohybrid materials consisting of graphene, polyethyleneimine (PEI), and AuNPs. The direct electron transfer of the GOx/graphene/PEI/Au-modified electrode was confirmed by the increase in anodic peak current and decrease in cathodic peak current observed during CV analysis with the addition of glucose conducted in PBS electrolyte solution with the absence of O_2_. The GOx/graphene/PEI/Au-modified electrode produced high sensitivity and low LOD of 93 μA mM^−1^ cm^−2^ and 0.32 μM, respectively, in the linear detection range of 1 and 100 μM. Direct electron transfer was achieved due to strong covalent binding from the cross-linking reaction of the GOx enzyme with the -NH_2_ group of PEI that facilitated electron transfer.

Interesting work has been conducted by Chu et al. [143] on developing in-situ synthesis of thiol (-SH)-grafted graphene nanomaterials and Au NCs (AuNCs) for immobilization of GOx enzyme. The presence of -SH group on the graphene nanomaterials causes GOx enzymes to be immobilized directly without the addition of a cross-linker. Interestingly, this work reported the effect of deposition potential and electric quantity on the morphology and size of the Au nanocrystal produced on the graphene/Au-modified electrode. Various forms of morphology influence the electrocatalytic behavior of the modified electrode during glucose detection. The -SH group on graphene can control the growth behavior of Au nanocrystal. A uniform Au nanocrystal was formed due to strong interaction between the -SH group and Au electrode.

**Figure 10:**
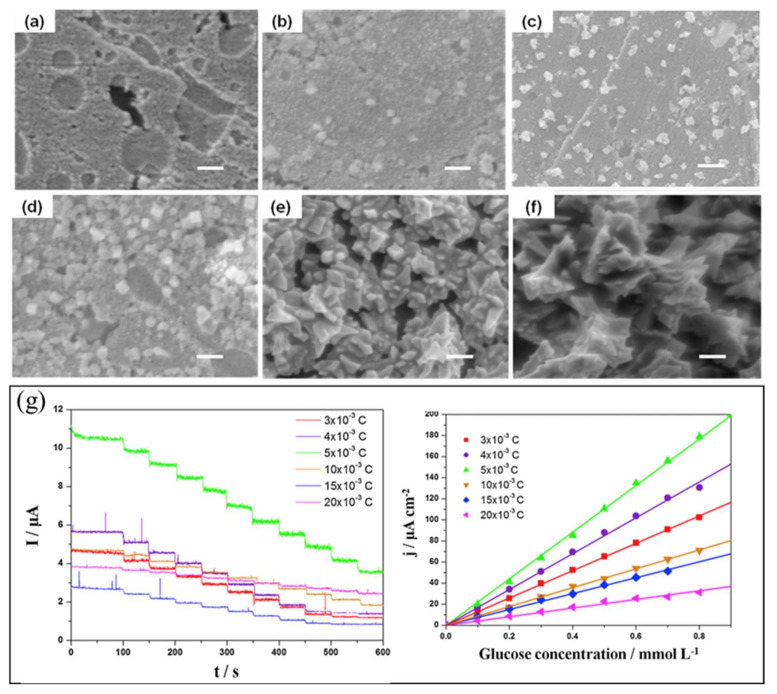
SEM images of Au nanocrystal deposited on the graphene/Au disk electrode under the same potential of −0.2 V with varied electrical quantities: (**a**) 2 × 10^−3^ C; (**b**) 3 × 10^−3^ C; (**c**) 4 × 10^−3^ C; (**d**) 5 × 10^−3^ C; (**e**) 10 × 10^−3^ C; and (**f**) 15 × 10^−3^ C. (**g**) The current signal of the amperometry response of the GOx/AuNC/graphene/Au disk electrode with varying electrical charges. Reprinted with permission from ref. [143]; Copyright 2015 Elsevier.

Figure 10a–f show the scanning electron microscopy (SEM) images of the Au nanocrystal electrodeposited on graphene, with varying electric charges of 2 × 10^−3^ C, 3 × 10^−3^ C, 4 × 10^−3^ C, 5 × 10^−3^ C, 10 × 10^−3^ C, and 15 × 10^−3^ C, respectively. Figure 10g shows the chronoamperometry response of the GOx/AuNC/graphene/Au-modified electrode with varying electrical charges. At low electric charge, the morphology of Au nanocrystal deposited on graphene at certain spots, leaving a high surface area of graphene exposed (Figure 10a,b). Therefore, only low current signal in amperometry analysis was observed. When the electric charge further increased to 4 × 10^−3^ C, more Au nanocrystals deposited on graphene were observed (Figure 10c), and some were in cubic shape (Figure 10d). At 5 × 10^−3^ C electric charge, the Au nanocrystals with cubic shape demonstrated complete growth. With better morphology and AuNC structure, high current signal in amperometry analysis was observed for the electrode deposited at 4 × 10^−3^ C and 5 × 10^−3^ C electrical charge. When the electrical charge was further increased to 10 × 10^−3^ C and 15 × 10^−3^ C, Au nanocrystal stacking formed a dense film with flower-like structure (Figure 10e,f). The dense structure and thick film thickness produced lower current signal in amperometry analysis due to the decrease in catalytic area during glucose detection.

A glucose biosensor based on the GOx/graphene oxide/AuNP/graphite electrode was recently developed [144]. The modified electrode exhibited direct electron transfer with a low LOD of 1.2 μM. The interconnection between the GO nanosheet and AuNPs facilitated an effective electron transfer pathway from active FAD of the GOx enzyme to the graphite electrode. Cai et al. [145] recently proposed three GOx models to represent the direct electron transfer of GOx on poly(3,4-ethylene dioxythiophene): poly(styrene sulfonate) (PEDOT:PSS) hydrogel-decorated carbon nanotube fiber (CNTF) electrode. The possible mechanism of direct electron transfer is through coating of the GOx enzyme with PEDOT:PSS hydrogel nanofibers, which created a tunnelling pathway between the FAD active GOX enzyme and CNTF electrode. The GOx/PEDOT:PSS/CNTF electrode exhibited sensitivity of 43.52 μA mM^−1^ cm^−2^ and linearity of 0.05–0.5 mM. The three GOx conditions proposed were deactivated and direct electron transfer-enabled GOx, enzyme catalytic and direct electron transfer-disabled GOx, and enzyme catalytic direct electron transfer-enabled GOx. Among all the three models explained, the model based on enzyme catalytic direct electron transfer-enabled GOx best represented direct electron transfer on the developed modified electrode.

**Table 3:** Summary of enzymatic glucose biosensors based on metal-based nanomaterials and nanohybrid/nanocomposite.

Electrode Modification	Nanomaterials Modified Electrode	Enzyme/ Immobilization Technique	Applied Potential	Linearity (mM)	Sensitivity (µA mM^−1^ cm^−2^)	LOD (µM)	Stability/Lifetime	Sample	Reference
AuNP-GOx-AuNPs/ODA-Pt	Langmuir–Blodgett deposition	GOx-Adsorption	0.60 V	0.1–5	0.52	63	95% 6 month	-	[127]
AuNP/GOx/ODA-Pt	Langmuir–Blodgett deposition	GOx-Adsorption	0.60 V	0.1–5	0.36	59	95% 6 month	-	
GOx/AuNPs/Pt/ODA-Pt	Langmuir–Blodgett deposition	GOx-Adsorption	0.60 V	0.1–5	0.31	59	95% 6 month	-	
GOx/ODA-Pt	Langmuir–Blodgett deposition	GOx-Adsorption	0.60 V	0.1–5	0.21	7	95% 6 month	-	
Nafion/GOx/AuNPs/OPPy/Au-PLA-MNs	Electrodeposition	GOx-Crosslink (GA)	0.75 V	0–2.6	8.09	40	14 days	-	[134]
GOx/AuNPs/PHCQE-Graphite	Electropolymerization	GOx-Crosslink (GA)	–0.70 V	0.75–3.125	0.13	17	42 days	Beverage	[136]
Nafion/GOx-TCA/Au Microneedle	Electropolymerization	GOx-Covalent (EDC/NHS)	0.45 V	0–22.2	0.22	19.4	94% 30 days	Human Serum	[135]
PPy/GOx/AuNPs/ Graphite Rod	Electrodeposition	GOx-Crosslink (GA)	0.30 V	0–19.9	21.70	200	9.8 days	Human Serum	[139]
GOx-SAM/Dendritic Au Nanostructure/ Graphite rod	Electrodeposition	GOx-Covalent (EDC/NHS)	0.30 V	0.1–10	-	19	73.25% 12 days	Human blood glucose	[105]
GA-GOx/Dendritic Au Nanostructure/Graphite rod	Electrodeposition	GOx-Crosslink (GA)	0.30 V	0.1–10	-	22	66.20% 12 days	Human blood glucose	
GOx/3D Au/carbon paper	Electrodeposition	GOx-Covalent (EDC/NHS) and Crosslink (GA)	0.25 V	0.002–21.97	96.27	0.6	80% 30 days	Human serum	[140]
GOx/PANI hydrogel/Pt	Chemical reduction	GOx-Crosslink (GA)	0.56 V	0.01–8	96.1	0.7	-	-	[133]
GOx-PoPD/PtNPs/ PVF + ClO_4_^−^/Pt	Electrodeposition	GOx-Electropolymerization	0.60 V	0.06–9.64	17.4	18	95% 15 days	Blood serum sample	[137]
GOx-PtNPs-PAA-aSPCEs	PtNPs-Electrodeposition PAA-Elctropolymerization	GOx-Adsorption	0.20 V	0.02–2.3	42.7	7.6	50% 7 days	Commercial juices	[138]
GOx/Pt film/o-phenylenediamine-ß-cyclodextrin/Au	Electrodeposition	GOx-Electropolymerization	0.25 V	2.5–15	111.21	0.75 mM	93.22% 4 days	Human serum sample	[146]
PU-PEG/GOx/Pt film/Au-PET	Electroplatting Pt film	GOx-Crosslink (GA)	0.65 V	0.5–25	3.418	0.25	90% 23 days	Beverage	[147]
GOx/Pt/rGO/poly(3-aminobenzoic acid/SPCE	Co-Electrodeposition	GOx-Covalent (EDC, NHS)	0.50 V	0.25–6.00	22.0	44.3	86% 7 days	Serum sample	[148]
Nafion/GOx/PtNP-CGr-f@MWCNTs /Au	Pt-Electrodeposition CGr-F@MWCNT-Drop Casted	GOx-Covalent (EDC, NHS)	0.50 V	0.005–13	26.5	5	21 days	-	[149]
Nafion/GOx/Graphene/ PtNPs	Drop Casted	GOx-Crosslink (GA)	0.60 V	0.005–0.5	-	0.01	75.45% 31 days	Human serum sample	[150]
GOx/Fc-bPEI-AuNPs/GCE	Drop Casted	GOx-Crosslink (GA)	0.43 V	0.5–10	800	0.04	-	-	[151]
GOx/PPy/AuNPs/SP-Graphene Ink-PET	Electrodeposition	GOx-Electropolymerization	0.40 V	1–10	14.453 nA/mM	-	90% 30 days	-	[122]
Nafion/GOx/Au–Ni coaxial nanorod array/Au electrode	Nano electroforming and immersion gold	GOx-Adsorption	0.40 V	0.028–27.5	778.2	5.5	87% 30 days	-	[152]
Nafion/GOx/Pd-MWCNT-SPCE Bulk	MWCNT-CVD Impregnate Pd	GOx-Adsorption	–0.20 V	0.41–4.12	–6.36	0.02	14 days	Human blood glucose	[95]
Nafion/GOx/Pd-MWCNT/SPCE	MWCNT-CVD Impregnate Pd	GOx-Absorption	–0.20 V	0.41–4.12	–5.05	0.14	14 days	Human blood glucose	
GOx-Graphene-PEI-AuNPs/Au Electrode	Microwave-irradiation	GOx-Crosslink (GA)	–0.35 V	0.001–0.1	93	0.32	88% 10 days	Human Serum	[111]
GOx-Graphene-Thiol/Au Nanocube/ Au disk	Au Nanocube-Electrodeposition	GOx-Adsorption	–0.40 V	0–0.8	221.0	-	79.3% 14 days	-	[143]
GOx-Chitosan/rGO-AuNPs/	rGO-AuNPs- Drop Casted	GOx-Covalent (Chitosan)	–0.30 V	0.1–1.3	34	76	70% 36 days	-	[153]
GOx/AuNPs/PENDI/PGE	AuNPs-Electrodeposition PENDI-Electropolymerization	GOx-Adsorption	-	0.0009–0.33	0.172	0.0407	-	-	[154]
Nafion/GOx/Carbon Fibre-Hemain AuNP/ Graphite Electrode	Carbon Fibre-AuNPs- Drop Casted	GOx-Nanoenzyme	–0.10 V	0.1–0.9	909.5 A⋅M^−1^⋅m^−2^.	0.05	-	Beverage	[155]
Au@rGO/PIn/Ferritin/GOx/GCE	Electrodeposition	GOx	-	50	7.2 mA cm^−2^	-	-	-	[156]
Graphite NPs-Pyrene-GOx/GCE	Drop Casted	GOx-Crosslink (pyrenebutyric-NHS)	0.60 V	0–2.2	7.29 × 10^−2^ nA	50	30 days	Urine	[157]
GOD-CS/AgNWs/GCE	Drop Casted	GOx-Covalent (Chitosan)	–0.15 V	0.01–0.8	-	2.83	83% 10 days	Human blood glucose	[158]

Abbreviations: ODA, octadecylamine; OPPy/Au-PLA-MNs, overoxidise polypyrole/gold-polylactic acid-microneedles array; PHCQE, polymerization dihydrothieno [3,4-b][1,4]dioxin-5-yl)-3-(9-hexyl-9H-carbazole-3-yl)quinoxalin-2-yl)-9-hexyl9H-carbazole monomer; TCA, terthiophene carboxylic acid; SAM, self-assembly; GA, glutaraldehyde; PoPD, polyvinyl fluoride and o-phenylenediamineand polyvinylferrocenium perchlorate; PMS, nmethylphenazonium methyl sulphate; PAA, poly(Azure A); aSPCE, activated SPCE; PU-PEG, polymerized indole, polyurethane-poly(ethylene glycol); PET, polyethylene terephthalate film; bPEI, polyethylenimine; PPy, polypyrrole; Sp, screen-printed; PENDI, polymerized N,N′-bis(2-hexyl)-2,6-(3,4 ethylenedioxythiophene)-1,4,5,8-naphthalenimide; PGE, pencil graphite electrode; AuCS, Au cylindrical spiral; f@MWCNTs, functionalized multiwalled carbon nanotubes; PIn, polymerization of indole; rGO, reduced grapheme oxide; CS, chitosan.

### 4.2. Metal Oxide-Based Enzymatic Glucose Biosensor

Despite the excellent properties of metal nanoparticles, they are expensive and exhibit low selectivity due to their small current response to target molecules. Recently, metal oxide nanostructured materials have gained interest as matrices for the development of glucose biosensors because of their unique physical, chemical, and catalytic properties [93]. Metal oxide nanostructured materials offer the advantages of good biocompatibility and non-toxic properties, good electrical conductivity, and relatively low production cost. Metal oxide nanoparticles with high surface area and high isoelectric point (IEP) offer high surface area for immobilization of low IEP enzyme biomolecules [49]. Therefore, good electrostatic absorption between metal oxide nanoparticles and enzyme biomolecules occurs at different charges on their surface. The high electrical conductivity of metal oxide nanoparticles is another interesting factor in amplifying the sensitivity of glucose biosensors due to the good electrical communication ability of the nanomorphological structure and FAD active center of the GOx enzyme biomolecules. Metal oxide nanostructured materials that are commonly used to modify glucose biosensor electrodes are zinc oxide (ZnO) [159,160], iron oxide (Fe_3_O_4_) [161,162,163], copper oxide (CuO) [164,165], cerium oxide (CeO_2_) [40,166], and manganese dioxide [167,168]. The advantages and disadvantages of common metal oxide nanostructured materials used in the modification of working electrodes for glucose biosensor applications are summarized in Table 4.

In self-assembly and layer-by-layer assembly, IONP or its composite in solution is immobilized onto the electrode. The benefit of the assembly method is that any type of nanomaterial structure and functional group can be applied for the modification of the electrode. However, the challenge of this method lies in the difficulty to control the morphology of IONPs on the electrode surface. Therefore, obtaining stable dispersion of IONPs in carrier liquid is important so that uniform morphology of IONPs on the modified electrodes can be obtained to ensure efficient enzyme immobilization on the matrix and reduce matrix interference. These rules are also applicable for immersion, where IONPs or their composites are spontaneously organized on the electrode surface [173]. Table 5 lists the enzymatic glucose biosensor metal oxide-based nanomaterials and nanohybrid/nanocomposite.

As for electrochemical deposition, uniform, thin layer, and good adhesion of IONPs or its composites can be deposited on the electrode surface. This was conducted by a simple electrolysis of a solution containing Fe ions or its chemical complex. This method offers better control over the morphology of the developed IONPs on the electrode, but the drawbacks of this method are the limitation of mass transport and limitation to functionalize the already developed IONPs or composites on the electrode [174].

Li et al. [175] reported Pt nanoparticle-decorated IONPs-MWCNT for the modification of glass carbon electrode (GCE) in glucose detection. The GOx enzyme was then physically absorbed on the nanocomposite (Pt/IONP-MWCNT/chitosan/GCE) and protected with Nafion film layer. The combination of IONP-MWCNT and Pt amplified the sensitivity and specificity of the developed glucose biosensor due to their high surface area, good mechanical stability, and good conductivity. The fabricated amperometric glucose biosensor had a broad linear range (6 µM–6.2 mM) and low detection limit (2 µM).

Li et al. [176] later reported the fabrication of a glucose biosensor based on one step-electrodeposition of IONP-AuNP-chitosan composite on the Au electrode. The electrodeposition method can control the film thickness of the nanocomposite present on the electrode. They found that the uniform IONP-AuNP-chitosan composite film allowed good immobilization of the GOx enzyme via physical absorption. The modified electrode (GOx/IONP-AuNP-chitosan/Au) showed great stability and good electrocatalytic activity toward glucose detection with linearity of 3 µM–0.57 mM and limit of detection of 1.2 µM.

Yang et al. [177] developed a glucose biosensor of Nafion/chitosan-IONP-GOx/Pt-modified electrode. The GOx enzyme was cross-linked with IONPs in chitosan medium using glutaraldehyde. The biosensor of Nafion/chitosan-IONP-GOx/Pt was fabricated through layer-by-layer assembly. A glucose biosensor with high sensitivity (11.54 μAmM^−1^ cm^−2^), low detection limit (6 µM), and wide linearity (6 µM–2.2 mM) was produced. This result was due to the properties of IONPs that could catalyze the H_2_O_2_ reaction, thereby amplifying the current response. They also claimed that their modified electrode was sensitive and specific for detecting glucose in human serum samples.

Peng et al. [31] employed physically entrapped GOx enzyme in polymerized nanocomposite of GOx-AuNP-polydopamine-IONPs for the GCE. The modified electrode has the magnetism of IONPs that makes them easily manipulated by an external magnetic field, excellent biocompatibility of polydopamine to maintain the native structure of GOx, and good conductivity of AuNPs to facilitate the direct electrochemistry of GOx in the biofilm. Thus, the presence of GOx-AuNP-polydopamine-IONP/GCE displayed good linear amperometric response to glucose concentrations ranging from 0.02 mM to 1.875 mM.

In our previous work, IONPs were used to modify the SPCE electrode [171] and ITO electrode [163] for a glucose biosensor. The IONPs were synthesized using the precipitation method and functionalized with citric acid to provide a hydrophilic surface and carboxyl (-COOH) functional groups for immobilization of the GOx enzyme. The Nafion/GOx/IONPs/SPCE modified electrode displayed good sensitivities for linear amperometric response of 175 and 5.31 μAmM^−1^ cm^−2^ to glucose concentrations ranges of 0.02–0.25 and 0.25–8.00 mM, with LOD of 7 µM. As for the Nafion/GOx/IONPs/ITO modified electrode, higher sensitivities of 995.57 and 5.8 μAmM^−1^ cm^−2^ for wide linear glucose concentrations ranging of 0.1–5.00 µM, and 5.0 µM–20.0 mM was developed. The IONPs with -COOH functionalization play an important role in providing a good biocompatible environment for GOx immobilization and enhanced binding capability for enzyme immobilization; thus, they facilitate electron transfer between GOx enzyme and the modified electrode.

ZnO nanostructure plays an important role by providing high surface area for GOx enzyme immobilization. Differences in ZnO nanostructure morphology significantly affect the glucose detection performance. Therefore, various morphologies of ZnO have been investigated as matrices for application in glucose biosensors. Among all nanostructure morphologies, 1D nanostructure is the most preferred matrix structure for ZnO. The 1D nanostructure offers several advantages such as high surface area-to-volume ratio and a controllable diameter of rods that are comparable with the size of an individual biological molecule. More analytes can be detected and eventually increase the sensitivity of the sensor. Moreover, the 1D ZnO nanostructure provides direct electron transport between electrode substrates and enzyme, which significantly improves the performance of biosensors.

Lei et al. [178] investigated the influence of different assembly processes of matrix onto the electrode on biosensor performance. In their work, they modified the Au electrode via directly grown ZnO NR array and transferred ZnO NR powder. For the transfer method, ZnO NRs were synthesized via hydrothermal method at 95 °C for 7 h, and the precipitate was collected and wetted with PBS solution before coating on the Au electrode. For direct growth, ZnO NRs were grown with a similar hydrothermal method on a horizontally immersed Au electrode. Both modified electrodes were immobilized with GOx enzyme and coated with a Nafion protective layer. A significant difference was found between the two conditions, where an enhanced sensitivity of 52%, fast response time, and low LOD were achieved for directly grown ZnO NRs compared with the transfer method sample. More GOx enzyme was immobilized on well-aligned NRs, which have higher surface area compared with randomly distributed and stacked ZnO NRs.

Ahmad et al. [179] studied the influence of NR surface area on the amount of enzyme immobilization and the performance of glucose detection. Different aspect ratios of ZnO NRs were directly grown on the Si/Ag electrode by varying the hydrothermal growth durations from 2 h to 14 h. By increasing the aspect ratio of ZnO NRs from 5 to 60, the immobilization percentage linearly increased and improved the sensitivity from 41.12 μA cm^−2^ mM^−1^ to 106.60 μA cm^−2^ mM^−1^. Nirmal and Swapan [180] also reported the effect of ZnO NR matrix aspect ratio on glucose detection performance by comparing different ZnO NRs at average lengths of 100 nm and 6 μm with a similar average diameter of 20 nm. They found that at a high surface area of ZnO NR matrix, a high sensitivity of 35.1 μA cm^−2^ mM^−1^ and wide linear range of 6.6 μM–0.38 mM were obtained compared with the small aspect ratio of ZnO NRs.

In another work, Fung et al. [181] fabricated an electrochemical sensor by using flexographic printing methods where three electrodes (working, counter, and reference) and a ZnO seed layer were selectively printed onto polyimide substrates. The zinc acetate precursor was prepared by mixing zinc acetate powder in deionized water and isopropanol, printing on the working carbon electrode, and annealing at 150 °C for 30 min to form a seed layer for hydrothermal growth. The modified substrate was subjected to continuous glucose sensing with an increment of 0.1 mM via chronoamperometry at an applied voltage of 0.8 V. The fabricated sensor exhibited a linear response to glucose concentration from 0 mM to 1.7 mM with a sensitivity of 1.2 ± 0.2 μA cm^−2^ mM^−1^ with calculated LOD of 46 ± 31 μM.

The hybridization and doping of ZnO with other nanostructures have been investigated to improve and enhance the performance of ZnO NRs as a transducer in glucose biosensors. Important advances have been achieved by combining and integrating nanocomposites as an individual component to enhance electron transfer and improve the performance of glucose biosensors. Several studies on ZnO hybrids with carbon-based materials [159,182,183] and metal-based materials [118,160,184] have been reported. Several studies have been carried out to produce a ZnO-graphite hybrid matrix for glucose biosensors due to the excellent properties of graphite. Gallay et al. [185] reported a fabrication of entangled ZnO nanowires (NWs) grown on compacted graphite flakes. As for glucose sensor fabrication, ZnO NW/graphite films were dispersed in water and filtered to form a colloidal suspension; subsequently, the supernatant colloid was mixed with GOx and left at 4 °C for 24 h for immobilization to occur. Finally, the immobilized ZnO NW/graphite solution was transferred to a Pt electrode and dried for 24 h. The electrode performance in glucose detection showed good sensitivity of 17 μA mM^−1^ cm^−2^ with low LOD of 9 μM.

The incorporation of metal nanoparticles onto the ZnO nanostructure surface can enhance the glucose sensor performance due to the excellent catalytic properties of metal nanoparticles for glucose to be oxidized. Moreover, as a result of their magnetic nature, metal nanoparticles can attract the immobilization of GOx on the matrix surface, which can enhance the glucose detection signal [186]. Zhao et al. [187] studied an enzymatic glucose biosensor based on ZnO NRs decorated with gold nanoparticles (AuNPs) that were synthesized by photo reduction from HAuCl_4_. The surface of ZnO NRs was modified with AuNPs via electrostatic adsorption, and the ZnO NR/FTO glass electrode was immersed in photoreduction solution and irradiated for 15 min under ultra-vitalux lamp. In their work, ZnO NRs were uniformly distributed on the FTO electrode with an average length of 2.5 μm, and the average diameter of AuNPs was 8–10 nm. Good sensitivity glucose detection was observed for the AuNP/ZnO/FTO (43.7 μA/mM cm^2^) electrode compared with the ZnO/FTO electrode (24.3 μA/mM.cm^2^). The surface-to-volume ratio of the matrix increased for higher GOx immobilization, and the good electrocatalytic ability of AuNPs facilitated the glucose oxidation/reduction process.

Chou et al. [184] studied the effect of AuNPs hybridized with ZnO NRs on the glucose biosensor performance. The ZnO NR/ITO electrode was functionalized with (3-mercaptopropyl) trimethoxysilane (MPTMS) before soaking in AuNP colloidal solution between 9 and 18 h. MPTMS was used to ensure that the anchoring of AuNPs on the rod surface was stable during immobilization and upon utilization. An intermediate layer consisting of the terminal functional groups of MPTMS was used to anchor the AuNPs either by electrostatic or chemical reaction. The results showed that the GOx/AuNP/ZnO/ITO-modified electrode had high catalytic activity and high sensitivity in glucose analyte compared with the bare GOx/ZnO/ITO electrode. They also compared the AuNP/ZnO/ITO electrode with and without GOx immobilization and found an increase in the separation peak (ΔEp) for the GOx/AuNP/ZnO/ITO-modified electrode. The reason could be attributed to low affinity between the GOx enzyme on the electrode and reduced electron transfer rate.

Besides AuNPs, platinum nanoparticles (PtNPs) have been incorporated onto the surface of ZnO NRs to enhance the sensor performance. Anusha et al. [170] prepared an enzymatic glucose sensor by dispersing PtNPs over ZnO by doctor-blading the commercial Pt paste. The modified electrodes were then subjected to chitosan (CS) coating as a protective layer and as bio-adhesion material to promote the electron transfer kinetics. They obtained a high sensitivity of 62.14 μA cm^−2^ mM^−1^ and low detection limit of 16.6 μM for the GOx/CS/Pt/ZnO/FTO electrode compared with the electrode without PtNPs on the ZnO surface. The presence of PtNPs on the ZnO surface enhanced electron transfer during re-oxidation of GOx, which resulted in high sensitivity. Previously, we developed a glucose biosensor based on ITO electrode modified with ZnO NRs decorated with Pt nanodendrite [169]. ZnO NRs were synthesized using a low-temperature hydrothermal technique, and Pt nanodendrite was synthesized through chemical reduction. The GOx enzyme was drop-casted on the Pt nanodendrite/ZnO nanorod/ITO-modified electrode and covered with Nafion layer to prevent mechanical interference from other electroactive molecules. The Nafion/GOx/42 nm Pt nanodendrite/ZnO NR/ITO-modified electrode showed sensitivity of 5.85 µA mM^−1^ with linear detection range within 1–18 mM and LOD of 1.56 mM.

Li et al. [188] reported the performance of an enzymatic glucose biosensor based on silver (Ag)-doped ZnO NRs with different silver nitrate (AgNO_3_) concentrations (0.023–1 mmol). They observed that an increase in reduction current reached a maximum value for silver content of 0.25 mmol and decreased subsequently. A high concentration of AgNO_3_ (0.6 and 1 mmol) decreased the effective surface area of the matrix surface. When the amount of AgNO_3_ increased, the rods became shorter and AgNPs attached on the rods became larger and inhomogeneous, which influenced the amount of GOx immobilized. The GOx/Ag-ZnO hybrid NR/GCE-modified electrode exhibited two linearity ranges of 0.01–0.1 and 0.1–1.5 mM, sensitivity of 18.7 µA M^−1^ cm^−2^, and LOD of 5 μM.

Previously, we have developed the direct growth of ZnONRs on a seeded ITO electrode via the hydrothermal method for a glucose biosensor [189]. The homogenous and spherical ZnO seed layer on the ITO electrode having 85 nm average diameter size has undergone an annealing process for 4 H at 500 °C, thus producing ZnONRs with an average diameter and length of 109.9 and 645.4nm, respectively. The GOx enzymes were immobilized on the ZnONRs/ITO modified electrodes and covered with a Nafion layer for glucose detection. The Nafion/GOx/ZnONRs/ITO modified electrodes exhibited good linearity towards glucose detection within the range of 0.05–1.00 and 1–20 mM with sensitivities of 48.75 and 3.87 µA M^−1^ cm^−2^, respectively. The excellent performance of the Nafion/GOx/ZnONRs/ITO modified electrodes in glucose detection are attributed to ZnONRs providing high surface area for GOx immobilization through electrostatic binding and the excellent conductivity of ZnO for facilitating fast electron transfer between GOx enzyme and the ZnONRs/ITO modified electrode. The reliability of the Nafion/GOx/ZnONRs/ITO modified electrode was tested using real blood samples and showed comparable performance to blood glucose level analyzed using commercial glucometer.

Then, we developed 40 nm Platinum nanodendrites (PtNDs) decorated on the ZnONRs/ITO modified electrode for the enzymatic glucose biosensor. The Nafion/GOx/PtNDs/ZnONRs/ZnO/ITO modified electrode [190] exhibited sensitivities of 98.34 and 9.77 μA mM^−1^ cm^−2^ for a linear detection range of 0.05–1 and 1–18 mM. The excellent performance was attributed to the catalytic properties and morphology of PtNDs. Dendrite’s structure has a high surface area of the electrode, which allowed more GOx enzyme to be immobilized and preserved the activity of enzyme.

**Table 5:** Summary of enzymatic glucose biosensors metal oxide-based nanomaterials and nanohybrid/nanocomposite.

Electrode Modification	Nanomaterials Modified Electrode	Enzyme/ Immobilization Technique	Applied Potential	Linearity (mM)	Sensitivity (µA mM^−1^ cm^−2^)	Stability/ Lifetime	LOD (µM)	Sample	Reference
GOx-PVA-IONPs/Sn	Drop-casted	GOx-Adsorption	–0.19 V	0.005–30	9.36	81% 30 days	8	-	[96]
Nafion/GOx/Pt/IONPs-MWCNTs-CS/MGCE	Electrodeposition	GOx-Adsorption	0.30 V	0.006–6.2	-	86.8% 14 days	2	-	[175]
Nafion/GOx/Nafion-IONPs@SiO_2_-MWCNT/GCE	Drop-casted	GOx-Adsorption	0.10 V	0.001–30	-	-	0.8	-	[97]
GOx/IONPs-AuNPs-CS/Au	Electrochemical deposition	GOx-Adsorption	–0.40 V	0.003–0.57	-	82.6% 14 days	1.2	-	[176]
Nf/CS-IONPs-GOx/Pt	Drop-casted	GOx-Crosslink (GA)	0.40 V	0.006–2.2	11.54	84% 30 days	6	Human Serum	[177]
Nf-GOx-HRP/AuNPs-IONPs@SiO_2_/ITO	Drop-casted	GOx-Crosslink (GA)	–0.20 V	0.05–1.0 1.0–8.0	92.14 15.00	94.8% 30 days	10	Human Serum	[191]
GOx/AuNPs/BSA-IONPs/Pt	Immersed	Covalent GOx-BSA	0.40 V	0.25–7.0	115.13	81% 30 days	3.54		[112]
GOx/IONPs/CS-Graphene/Pt	Drop-casted	Gox- Covalent (EDC/NHS)	0.50 V	0–26	5.658	75.7% 30 days	16	-	[192]
GOx-Au-PDA-IONPs/MGCE	Drop-casted	Co-polymerization GOx	–0.50 V	0.02–1.875	-	95% 30 days	6.5	Human Serum	[31]
GOx/rGO-IONPs/MSPCE	Drop-casted	Electrostatic interaction GOx	–0.45 V	0.05–1.0	5.90	95.1% 30 days	0.1	-	[142]
GOx-IONPs@AuNPs/MnO_2_-SPCE	Drop-casted	GOx-Adsorption	0.38 V	0.2–9.0	2.52	80% 30 days	13.2	Beverage	[193]
Nafion/GOx/IONPs-CA/SPCE	Drop-casted	GOx-Adsorption	–0.43 V.	0.02–0.25 0.25–8.00	175 5.31	60% 30 days	7	-	[171]
Nafion/GOx/IONPs-CA/ITO	Drop-casted	GOx-Adsorption		0.0001–0.005 0.005–20.0	995.57 5.81	-		-	[163]
L-Cys/GOx/PVA/ ZnO/Au	Sputtered Deposition and Direct Growth	GOx-Adsorption	0.06 V	0.25–19	70.2	94% 45 days	1	Urine	[179]
GOx/ZnO Nanotube/AuCS	Hydrothermal Direct Growth	GOx-Adsorption	0.80 V	0–6.5	2.63	80.8% 20 days	8	Human Serum	[194]
GOx/ZnO Nanowire/ Carbon/Polymide	Flexographic printing	GOx-Adsorption	0.80 V	0–1.7	-	-	1200	-	[181]
Nafion/GOx/ZnO Nanorod/Zn foil	Hydrothermal Direct Growth	GOx-Adsorption	0.50 V	0.006–0.38	35.1	-	-	-	[180]
Nafion/GOx/ZnO/Au	Drop-casted	GOx-Adsorption	0.80 V	0.01–5.9	23.43	-	10	-	[178]
Nafion/GOx/ZnO Nanorod/Au/ SiO_2_-Si	Hydrothermal Direct Growth	GOx-Adsorption	0.40 V	1–10	315	89% 11 days	166.6	-	[195]
GOx/ZnO Nanowire/Au-PET	Electrodeposition	GOx-Adsorption	0.80 V	0.2–2	19.5	-	50	-	[196]
GA-GOx/rGO-Fc/GCE	Drop-casted	GOx-Crosslink (GA) Ferrocene	0.35 V	2–10	-	70% 14 days	0.02	Human Serum and Juice	[197]
GOx/rGO-Fc(COOH)_2_/GCE	Drop-casted	GOx-Adsorption -COOH F(x)	0.35 V	1–10	-	70% 14 days	0.04	Human Serum and Juice	
GOx-SiO_2_/Lig/Fc/CPE	Casted	Gox absorption with ferrocene mediator	0.60 V	0.5–9	0.78	73% 21 days	145	Liquid Glucose	[114]
Au@rGO/PIn/Ferritin/GOx/GCE	Electropolyme-rization	GOx-Elctropolymeri zation	LSV	50	7.2 mA cm^−2^	-	-	-	[156]
Graphite NPs-Pyrene-Gox/GCE	Drop-casted	GOx-Crosslink (pyrenebutyric acid/NHS)	0.60 V	0–2.2	7.29 × 10^−2^ nA	30 days	50	Urine	[157]
GOx-CS/AgNWs/GCE	Drop-casted	GOx-CS	-0.15 V	0.01– 0.8	-	83% 10 days	2.83	Human Blood Glucose	[158]
Nafion/GOx/Fe_3_O_4_ /ZnONFs/Au/PET	ZnO-Hydrothermal Direct Growth Fe_3_O_4_-Drop-casting	GOx-Adsorption	0.80 V	0.089–12.5	4.52	-	0.089	Human Blood Glucose	[161]
Nafion/GOx/ZnO Nanoflower/Au/PET	Hydrothermal Direct Growth	GOx-Adsorption	0.80 V	0.15–8.5	0.57	-	0.105	Human Blood Glucose	[161]
Nafion/GOx/Pt Nanodendrite/ZnO NR/ITO	Hydrothermal Direct Growth and Spin-coated	GOx-Adsorption	-	1–18	5.85	-	1.56	-	[169]
Nafion/ZnONR/ITO	Hydrothermal Direct Growth	GOx-Adsorption	–0.5 V	0.05–1 1–20	48.75 3.87	85% 14 days	-	Human Blood Glucose	[189]
Nafion/GOx/AuNP/ ZnONR/ITO	Hydrothermal Direct Growth and Drop-casted	GOx-Adsorption	–0.5 V	0.05–1.0 1.0–20	14.53 2.54	-	-	-	[198]
Nafion/GOx/PtNDs/ZnONRs/ITO	Hydrothermal Direct Growth and Drop-casted	GOx-Adsorption	–0.5 V	0.05–1 1–18	98.34 9.76	76.1% 30 days	0.03	Human Blood Glucose	[190]
Nafion/GOx/Au/ZnONRs/ITO	Electrodeposition	GOx-Adsorption	0.80 V	0–20	20.19	-	0.5	-	[118]

Abbreviations: PVA, polyvinyl alcohol; AuCS, IONPS, iron oxide nanoparticles; Cs, chitosan; MWCNTs, multiwalled carbon nanotube; MGCE, magnetic glass carbon electrode; PDA, polydopamine; rGo, reduced graphene oxide; MSPCE, magnetic screen-printed carbon electrode; L-Cys, L-cystene; Lig, Lignin; Au cylindrical spiral; Pin, polymerization indole; Fc(COOH)2,ferrocene dicarboxylic acid; PET, polyethylene terephthalate; ZnO NR, zinc oxide nanorods.

## 5. Recent Development of Nanomaterial-Modified Electrode for Non-Enzymatic Glucose Biosensor

As mentioned earlier, the enzymatic glucose detection method relies on the catalytic reaction of GOx enzyme to oxidize glucose. In non-enzymatic glucose biosensors, the electrode surface serves as the catalyst for the electrooxidation of glucose to occur. The non-enzymatic glucose biosensor eliminates the dependence of biological components for the glucose oxidation reaction. In general, there are two widely accepted models that explain the electrooxidation of glucose in non-enzymatic glucose biosensors: the activated chemisorption model and the incipient hydrous oxide adatom mediator (IHOAM) model [35,59].

**Figure 11:**
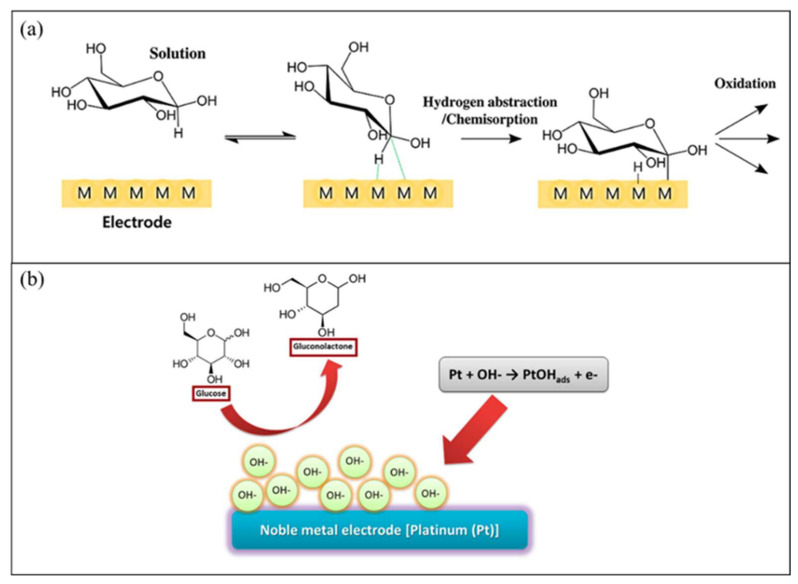
Schematic of (**a**) chemisorption model. Reprinted with permission from ref. [200]; Copyright 2018 Elsevier. (**b**) IHOAM model of glucose oxidation. Reprinted with permission from ref. [35]; Copyright 2021 Frontiers.

The activated chemisorption model was proposed by Pletcher (1984) [199], and this model involves the adsorption–desorption of glucose molecules on the electrode surface. Figure 11a shows the schematic of glucose oxidation in the chemisorption model. The chemical interaction between C-1 and the hydrogen atom of glucose molecules increases as the glucose molecule moves toward the electrode surface, which causes C-1 to dehydrogenate and adsorb on the electrode surface. Subsequently, when the electrooxidation of adsorbent occurs, gluconolactone is oxidized into gluconic acid through various pH-dependent routes. A suitable geometry of the electrode, which provides space for adsorption sites, controls the kinetic enhancement of the glucose oxidation process and the electrocatalyst interaction with the adsorbent. Other factors that influence the adsorption–desorption of glucose molecules are the electronic state of the redox center, unoccupied d-orbitals at transition metal centers, and defects in the non-metallic based catalyst [200].

The second model, known as the IHOAM model, was proposed by Burke (1994). The IHOAM model specifies the role of hydroxyl radicals in the electrocatalytic process. This model is based on the presence of active metal atoms, which involve a pre-monolayer oxidation step, forming the incipient hydrous oxide (OH_ads_) layer. The OH_ads_ layer on the metal-electrode surface helps facilitate the oxidation of glucose [201]. The chemisorption of OH_ads_ on the reductive metal-electrode adsorption sites causes the formation of MOH_ads_, which then oxidizes the glucose molecules. Figure 11b shows an illustration of the IHOAM model. Both the proposed models on chemisorption and IHOAM are based on noble metal electrodes only. The catalytic process of glucose oxidation in metal oxide electrodes is based on anodic bias, where the metal oxide layer with a low oxidation number is oxidized into metal oxide with a high oxidation number. The high oxidation number metal oxide has great ability to create the hydrous oxide (OH_ads_) layer, which then mediates the glucose oxidation process. As for the transition metal electrode, the redox center of the transition metal plays a role in the glucose oxidation reaction. The suitable electrolyte condition for the non-enzymatic glucose biosensor is either alkaline or neutral conditions. The high affinity of the hydrous oxide (OH_ads_) layer forms at alkaline conditions. The acidic electrolyte condition is not suitable due to the instability of transition metal and metal oxide-based electrode materials.

Glucose detection in non-enzymatic glucose biosensors is dependent on the electrode materials, aspect ratio, morphology structure, active site energy, and catalytic activity of the electrode surface [35]. In non-enzymatic glucose biosensors, noble metal working electrodes such as Pt and Au electrode are normally applied for glucose detection. However, the practical limitation of noble metal electrodes lies in the slow kinetics for the glucose catalytic reaction caused by the presence of other biological components in blood and the toxic effect caused by chloride ions or intermediate products generated during glucose catalysis. The integration of nanomaterials in the modification of electrodes in non-enzymatic glucose biosensors significantly improves the catalytic activity during glucose detection. Therefore, nanostructure material-modified non-enzymatic glucose biosensors based on metal, metal oxide, carbon, or their composite/hybrid nanomaterials have been widely explored to produce effective detection of glucose with high sensitivity, wide linearity, and low LOD.

### 5.1. Metal-Based Non-Enzymatic Glucose Biosensor

Apart from alkaline metals, most metals have excellent electrical conductivity properties due to metallic bonding. In non-enzymatic glucose biosensors, the electrode is commonly modified with noble metals (Pt, Au, and Pd) and transition metals (Co, Ni, and Cu). Table 6 lists the advantages and disadvantages of metal nanomaterials for the modification of electrodes for non-enzymatic glucose biosensors. To further enhance the electrocatalytic performance of non-enzymatic glucose biosensors, porous and rough structure, high-index crystalline facet, high-aspect-ratio nanostructure materials, and high-oxygen vacancy nanomaterials have been employed in the modification of electrodes for glucose biosensors.

Porous nanostructure materials provide a high surface-to-volume ratio for the adsorption of glucose molecules, thereby enhancing the catalytic performance. In porous structures, the surface roughness determines the electrochemical activity. High surface roughness leads to high electrochemical activity. Previously, Xu et al. [208] developed nanoporous Pt to modify GCE, which exhibited good linearity of 0.1 µM to 8.13 mM and LOD of 7.75 µM for glucose detection in neutral pH conditions. Nanoporous Pt was prepared through a two-step process, which involved electrodeposition of Pt–Cu alloy on the GCE and selective anodic dissolution of Cu in the alloy by electrochemical method. The 3D nanoporous Pt, with a pore size in the range of 80–150 nm and surface roughness factor of 184, provided additional sites for glucose molecule adsorption, which enhanced the catalytic performance. The modified electrode was successfully applied in measuring the glucose level in blood samples. The non-enzymatic glucose biosensors based on metal-based nanomaterials, metal oxide-based nanomaterials, and nanohybrid/nanocomposite are listed in Table 7.

A similar approach was reported by McCormick and McCrudden [203], where a nanoporous Pt-modified SPCE was developed through cyclic electrochemical deposition on Pt-Cu alloy, followed by subsequent electrochemical dealloying of Cu. During the cathodic scan, the Pt-Cu alloy was deposited, whereas Cu was dissolved during the anodic scan. The nanoporous Pt with surface roughness factor of 3680 was obtained. The nanoporous Pt/SPCE-modified electrode exhibited linearity of 1–13 mM in neutral PBS electrolyte (pH 7.4) with high selectivity against common interference molecules. Lee et al. [125] developed Pt nanopore-modified disposable screen-printed carbon on polyimide film (SPCE-polyimide). Pt nanopore was synthesized via chemical reduction in a reverse micellar phase. The mixture of Pt nanopore and binding polymer was used to modify SPCE-polyimide via the dispensing technique. The Pt nanoporous/SPCE-polyimide-modified electrode showed good linearity for glucose detection in a neutral (pH 7.4) PBS electrolyte condition.

**Figure 12:**
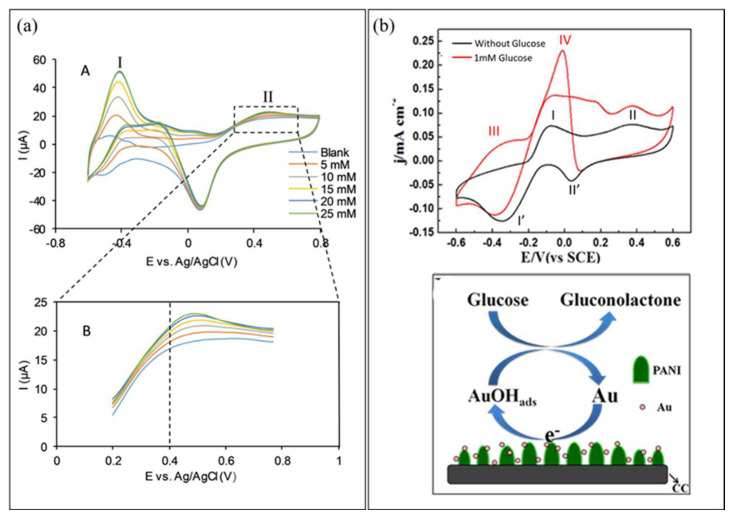
CV of (**a**) nanoporous Pt/SPCE−polyimide-modified electrode in the absence and presence of various glucose concentrations and the magnified area of glucose oxidation with 0.4 V oxidation potential in 0.1 M PBS (pH 7.4) solution. Reprinted with permission from ref. [203]; Copyright 2020 Elsevier. (**b**) AuNP/PANI/carbon cloth-modified electrode in the absence and presence of 1 mM glucose in 0.5 M KOH solution, and the schematic of electrocatalytic glucose mechanism. Reprinted with permission from ref. [209]; Copyright 2017 Elsevier.

The glucose oxidation mechanism of the Pt-based electrode can be explained via CV analysis in the absence and presence of various glucose concentrations. As shown in Figure 12a, the peak position I (−0.4 V) indicated the hydrogen region, which represented the dehydrogenization of glucose adsorption to the electrode surface. Peak position II (potential region of 0.4–0.8 V) indicated the double layer region, which represented the direct oxidation of glucose. The performance of the Pt-based electrode in glucose oxidation was determined by peak position II. Figure 12a shows that increasing the glucose concentration increased the anodic peak current in the potential range of 0.4–0.8 V.

In another work, a nanoporous Au-modified electrode was developed by electrochemical deposition on Au-Sn alloy, followed by electrochemical dealloying of Sn [204]. Nanoporous gold was randomly arranged with rough surface and diameter of 50–100 nm on the Au electrode. The nanoporous Au/Au-modified electrode showed high electrocatalytic performance for glucose oxidation in alkaline electrolyte solution, with a high sensitivity of 4374.6 μA cm^−2^ mM^−1^, good linearity of 2 μM to 8.11 mM, and low LOD of 0.36 μM.

AuNP-modified GCEs have been developed through seed-mediated growth [205]. The effects of immersion time and concentration of Au seed applied to the morphology and structure of AuNPs were examined. The immersion time showed no obvious effect on the Au nanoseed morphology, but higher concentration of Au nanoseed produced denser attachment of Au nanoseed on the GCE. High-density and well-dispersed AuNPs on the GCE promoted excellent electrocatalytic of the glucose oxidation reaction. The AuNP/GCE-modified electrode exhibited wide linearity of glucose detection with 0.1–25 mM, sensitivity of 87.5 μA cm^−2^ mM^−1^, and LOD of 0.05 M. In another work, AuNPs modified with ITO were reported. Additionally, the crystal plane of nanomaterials influenced the catalytic activities. Wang et al. [210] reported the electrodeposition of AuNPs with (111) facet to modify the ITO electrode. The AuNP/ITO-modified electrode exhibited high sensitivity of 23 μA cm^−2^ mM^−1^ for the linear detection range of 0–11 mM and LOD of 5 μM. The good electrochemical performance of the AuNP/ITO-modified electrode toward glucose oxidation was due to the presence of the uniform distribution of the Au (111) facet on the ITO electrode.

Xu et al. [209] presented a flexible electrode of a carbon cloth modified with PANI and AuNPs. The PANI arrays were grown on a flexible carbon cloth through electropolymerization, followed by the electrodeposition of AuNPs. PANI arrays with 100 and 200 nm diameter were observed, and AuNPs with 20 nm were deposited. The AuNP/PANI array/carbon cloth-modified electrode exhibited a high sensitivity of 150 μA cm^−2^ mM^−1^, linearity of 0.01–10 mM, and LOD of 3.08 μM. The flexible modified electrode is suitable for the development of wearable glucose biosensors.

The mechanism of a Au-based modified electrode was explained by Xu et al. [209]. Figure 12b shows the CV peak curve without and with the presence of 1 mM glucose for the AuNP/PANI/carbon cloth-modified electrode. Two pairs of redox peaks (I/I’ and II/II’) of the AuNP/PANI/carbon cloth-modified electrode without the presence of glucose were observed. The anodic peak I at −0.09 V indicated the adsorption of OH^−^ on the AuNPs forming Au(OH)_ads_, whereas the anodic peak II at 0.39 V indicated the oxidation of Au(OH)_ads_ to Au oxide. As in the cathodic region, the peak II’ at 0.06 V indicated the reduction of Au oxide and the peak I’ at 0.34 V indicated the reduction of Au(OH)_ads._ In the non-enzymatic glucose biosensor, the active Au(OH)_ads_ served as the catalyst for the glucose oxidation reaction.

After the addition of 1 mM glucose, the oxidation current for peaks I and II increased and the reduction current for peaks I’ and II’ decreased. Additionally, one new peak (peak III) at −0.40 V appeared during the anodic scan. Peak III represented the adsorption of glucose on the AuNP/PANI/carbon cloth-modified electrode and the formation of gluconolactone. In the cathodic scan, the Au oxide on the AuNP/PANI/carbon cloth-modified electrode decreased. Many Au(OH)_ads_ were reconstructed with the negative scan and served as the active surface site for glucose oxidation. Therefore, higher anodic current was observed (peak IV) at 0 V. Peak IV corresponded to the direct oxidation of glucose in the cathodic potential with adsorption-controlled process and exhibited fast electron transfer performance. A similar mechanism was proposed by Wang et al. [210] and Pei et al. [204], who employed AuNP/ITO-modified electrode and nanoporous Au/Au-modified electrode. Scholars have suggested that the peak with high anodic current value obtained during cathodic potential can suitably represent the electrochemical performance of the modified electrode in glucose detection.

Copper-based nanomaterials modified electrodes for non-enzymatic glucose biosensors offer good catalytic properties for glucose oxidation performance [211]. Choudhary et al. [212] developed the Cu NPs-Polyaniline nanocomposite-modified GCE electrode for glucose biosensors using an in-situ polymerization technique. The Cu NPs-polyaniline/GCE modified electrode exhibited sensitivity of 0.474 μA cm^−2^ mM^−1^, and linearity of 0.4–4 mM. The Cu NP binds with the nitrogen chain presence in polyaniline and serves as the catalytic centre for non-enzymatic glucose detection. In another work, Cu microspheroids and copper oxide (CuO) urchin fabricated using the electroplating technique were used to modify the laser-induced carbon electrode on the flexible meta-polyaramid (Nomex) sheets [213]. The Cu micro-spheroids/carbon-Nomex and CuO urchin/carbon-Nomex modified electrode exhibited high sensitivities of 250 and 320 μA mM^−1^ cm^−2^, and low LOD of 1.75 and 7.56 μM, respectively, for a linear detection range of 1 μM to 3.3 mM, respectively. This finding opens the possibility of the development of flexible and durable glucose biosensors, which are suitable for implementation in continuous blood glucose monitoring and microfluidic system.

Recently, a porous Cu layer was developed using a colloidal crystal templating technique on the SPCE [214]. The nanoporous Cu/SPCE modified electrode showed high sensitivity of 3411 μA mM^−1^ cm^−2^, and low LOD of 0.1 μM for two wide linearity ranges of 0.2–1.0 mM and 1.0–100 mM. The high surface area of the Cu nanoporous structure enables excellent electrochemical detection for non-enzymatic glucose biosensors. The Nanoporous Cu/SPCE modified electrode exhibited excellent repeatability and selectivity against uric acid and ascorbic acid interference. Additionally, the Cu nanoporous/SPCE-modified electrode was also able to provide good electrochemical detection for all saccharide (galactose, fructose, and sucrose) molecules. Therefore, a pre-separation step is necessary if quantification of individual saccharides is required in terms of their application in food and environmental analysis. The combination of two or more metallic elements for the modification of electrode for non-enzymatic glucose biosensors is interesting to further enhance the electrocatalytic performance in glucose detection. Bimetallic or trimetallic alloys of noble-noble metal [34,215] or noble-metal oxide nanomaterials [216,217] have been developed. The combination of bimetallic or trimetallic components synergistically improves the oxidative current of glucose and anti-interference ability of non-enzymatic glucose biosensors.

Pak et al. [206] developed a non-enzymatic glucose biosensor based on the modification of fluorine-doped tin oxide (FTO) electrode with bimetallic Ni nanostructure/Au nanoparticles. AuNPs were deposited on the FTO electrode through physical vapor deposition and thermal annealing. The Ni nanostructure was electrodeposited on the AuNP/FTO-modified electrode. The Ni nanostructure/AuNP/FTO-modified electrode exhibited two linear ranges for glucose detection of 5 μM–3.5 mM and 3.5–7 mM, high sensitivity of 893 μA mM^−1^ cm^−2^, and low LOD of 0.7 μM. The direct contact between the Ni nanostructure and AuNPs on the FTO electrode produced a synergistic effect, which facilitated rapid electron transfer during glucose detection. In another work, bimetallic Co-Ni in a prism nanostructure was employed in the modification of the ITO electrode for non-enzymatic glucose biosensor [218]. The modified electrode of Co-Ni/ITO exhibited very high sensitivity of 5024.4 μA mM^−1^ cm^−2^ for linear glucose concentration of 0–14.3 mM and excellent selectivity.

The catalytic properties of noble metal materials are highly dependent on the structure of the atomic surface. The high index facet and low-coordinated atom offer high active sites for electrocatalytic activities of glucose oxidation. Recently, Wang et al. [34] developed Pt-Pd ink for the modification of a Au electrode in a non-enzymatic glucose biosensor. The structure of Pt-coated Pd NCs was tuned by varying concentrations of Pt precursor to obtain high-index facet nanostructure materials with excellent electrocatalytic performance for the glucose oxidation reaction. Four nanostructures of Pt-coated Pd were fabricated: concave Pt-Pd, core-shell Pt-Pd, core-shell Pt-Pd with Pt Island, and stellated Pt-Pd. The average particle size and the intensity ratios of the (1 1 1)/(2 0 0) peaks for the nanostructure materials of Pt-Pd formed were as follows: 17.6 nm and 0.63 for concave Pt-Pd, 17.8 nm and 0.96 for core-shell Pt-Pd, 18.3 nm and 1.1 for core-shell Pt-Pd with Pt Island, and 21.5 nm and 1.6 for stellated Pt-Pd. The average particle size and the intensity ratio of the (1 1 1)/(2 0 0) peaks of the nanostructure increased with increasing Pt precursor concentration. Among all fabricated Pt-Pd nanostructures, the catalytic activity of the concave Pt-Pd showed excellent electrocatalytic performance for glucose oxidation with wide linearity of 2.4–10.6 mM, sensitivity of 11.06 μA·mM^−1^·cm^−2^, and LOD of 0.15 µM. These results were attributed to concave Pt-Pd nanostructure containing (210) and (950) high indexed facets, which exhibited great activity and tolerance toward high concentrations of glucose.

In another work, the catalytic performance of the core-shell Pd@Pt nanostructure with varying geometries to the glucose oxidation reaction was compared [219]. The core-shell Pd@Pt nanostructures with octahedral, rhombic dodecahedral, and nanocubic geometries were fabricated using the seed-mediated growth method and employed for the modification of the carbon electrode. The Pt outer shell was deposited on the Pd core particles in the epitaxial direction. When the comparison was conducted based on the electrochemical surface area, the Pd@Pt octahedral and Pd@Pt rhombic dodehedral exhibited higher activities compared with the Pd@Pt NC.

### 5.2. Metal Oxide-Based Non-Enzymatic Glucose Biosensor

Metal oxide nanomaterials such as Fe_3_O_4_, MnO_2_, CuO, ZnO, NiO, and Co_3_O_4_ offer properties of excellent catalytic ability, being inexpensive, and good stability in the modification of electrodes for non-enzymatic glucose biosensors. Most commonly, metal oxide nanostructure materials such as nanotube arrays, NR array, and NW arrays were employed in the modification of electrodes for non-enzymatic glucose detection [220,221]. These 1D structures offer advantages of high electrochemical surface area, prepare a direct channel for fast electron transport, and eliminate the possible aggregation of nanoparticles.

Research on non-enzymatic glucose biosensors based on iron oxide NR arrays was recently reported [222]. The iron oxide NR arrays were synthesized by the electrochemical anodization of iron foil, followed by in situ annealing, forming high-crystalline iron oxide NR arrays. The glucose-sensing performance of the iron oxide NR arrays was tested electrochemically and resulted in linearity of 0.5–3.7 mM and detection limit of 0.1 μM. The non-enzymatic iron oxide NR array glucose biosensor demonstrated high sensitivity, high stability, and ability to be applied in a real sample measurement with minimum interference. The high performance of the array was attributed to the improved electron transfer pathway and the cooperative electrochemical oxidation of glucose by Fe(III) and Fe(II) species.

Chen et al. [220] fabricated iron oxide nanotubes on the FTO electrode for glucose biosensor applications. The magnetite phases of iron oxide nanotubes showed high sensitivity to glucose response of 673.3, 71.2, and 9.58 μAmM^−1^ cm^−2^ for glucose linear ranges of 0.1 μM–0.125 mM, 0.125–1.0 mM, and 1.0–5.0 mM, respectively. The excellent electrochemical sensing performance of the developed iron oxide nanotube arrays was due to the massive transport channel in nanometer scale provided by the iron oxide nanotubes for glucose accessibility.

Most commonly, metal oxide nanomaterials were combined with other metal oxide nanomaterials or metal nanomaterials to enhance the electrical conductivity properties of the modified electrode. Zhang et al. [192] reported a multifunctional composite with a combination of IONPs and water-stable graphene functionalized with CS for glucose biosensor application. The IONP/graphene-CS/Pt-modified electrode exhibited good glucose detection response with a sensitivity of 5.658 μAcm^–2^ mM^–1^, detection limit of 16 μM, and linear detection range up to 26 mM glucose. The combination of IONP graphene-CS offers large active surface areas for enhanced electron transport with formation of 3D hybrids and excellent magnetic properties with catalytic activity, which are useful for the fabrication of electrochemical sensing devices.

Imran et al. [202] reported a nanocomposite-modified Au electrode for non-enzymatic glucose biosensor. The nanocomposite composed of platinum-doped carbon nitride (Pt-gC_3_N_4_), and ZnO was drop-casted on the Au electrode for glucose detection. Pt-gC_3_N_4_ was synthesized by simple pyrolysis, whereas ZnO was synthesized through chemical reduction. The Pt-gC_3_N_4_/ZnO/Au-modified electrode showed high sensitivity of 3.34 μAcm^–2^ mM^–1^ for a wide linear glucose detection of 0.25–110 mM and LOD of 0.1 µM. The applicability of the modified electrode was tested with human serum, blood, and urine. The modified electrode showed high stability and could be reusable for four times in whole blood glucose detection without a reduction in catalytic performance.

**Figure 13:**
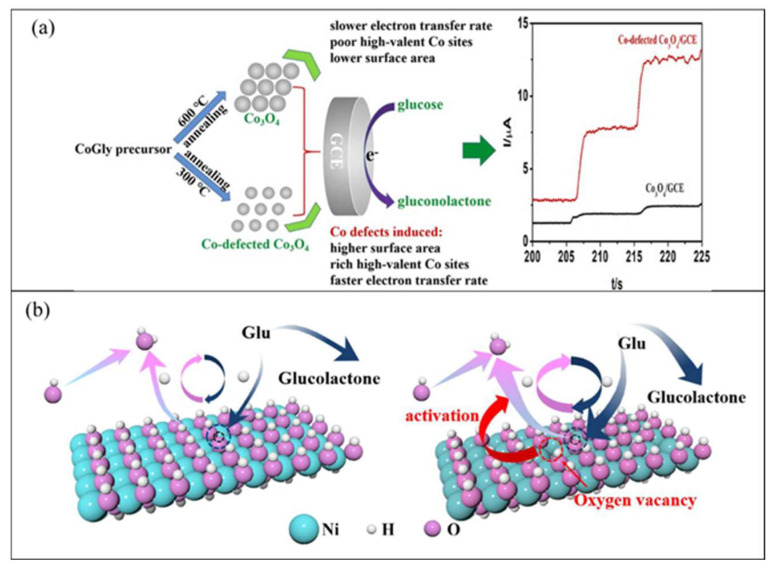
Schematic of (**a**) Co_3_O_4_ and Co-defected Co_3_O_4_ modification strategies and the amperometric performance with glucose addition. Reprinted with permission from ref. [207]; Copyright 2022 Elsevier. (**b**) Mechanism for glucose detection in pristine Ni(OH)_2_ and defect-Ni(OH)_2_. Reprinted with permission from ref. [224]; Copyright 2021 Elsevier.

Another possible way to improve the electrocatalytic properties of the modified electrode is through defect engineering. Through defect engineering, the electrical structure is modified, which exposes higher valence sites for glucose oxidation and facilitates the electron transfer rate. Very recently, Qi et al. [207] synthesized Co-defected Co_3_O_4_ by controlling the annealing temperature treatment of glycerolatocobalt (GlyCo) nanostructure. The Co-defected Co_3_O_4_ was used for modification of GCE, as shown schematically in Figure 13a. The Co-defected Co_3_O_4_/GCE-modified electrode exhibited linearity of 0.2 µM to 0.5 mM, low LOD of 0.16 µM, and high sensitivity of 2595.7 µA mM^−1^ cm^−2^ in glucose detection. The sensitivity of the Co-defected Co_3_O_4_/GCE-modified electrode was 10-fold higher than that of the normal Co_3_O_4_/GCE-modified electrode. The presence of metal defects in Co provided a bigger surface area rich with high-valent Co sites, thereby facilitating electron transfer during the glucose oxidation reaction.

In another work, the nanocomposite based on amorphous SnO_x_ decorated with CuO NRs was synthesized through hydrothermal method and calcined at 450 °C [223]. The nanocomposite of SnO_x_-CuO NRs was mixed with Nafion and drop-casted on GCE for the non-enzymatic glucose biosensor. The SnO_x_-CuO NR/GCE-modified electrode exhibited high sensitivity of 2303 µA mM^−1^ cm^−2^ for glucose detection in the range of 0.001–6 mM. The incorporation of amorphous SnO_x_ enhanced the oxygen vacancy defect in the nanocomposite. The oxygen vacancy defect encouraged the chemical adsorption of the glucose molecules and increased the dissociation of adsorbed molecules. Thus, the redox reaction rate increased, and the electrocatalytic activity of the SnOx-CuO NR/GCE-modified electrode was significantly enhanced.

Zhong et al. [224] utilized the defect Ni(OH)_2_ nanosheet for modification of nickel foam electrode through electrodeposition. Defects in the Ni(OH)_2_ nanosheet were created via Ar plasma treatment. The Ni(OH)_2_ nanosheet/Ni foam-modified electrode showed high sensitivity of 13,940 µA mM^−1^ cm^−2^ for glucose detection in the range of 0.001–0.5 mM. The activity of the glucose oxidation reaction in non-enzymatic glucose biosensors is dependent on the deprotonation reaction rate. A comparison of the glucose detection mechanism in pristine and defect Ni(OH)_2_ nanosheet is illustrated in Figure 13b. During glucose detection, Ni(OH)_2_ undergoes deprotonation, forming the intermediate NiOOH. The intermediate NiOOH acts as catalyst for the oxidation of glucose to gluconolactone, returning into Ni(OH)_2_. If the transformation of NiOOH into Ni(OH)_2_ occurs rapidly, then the accumulation of NiOOH intermediate can be prevented. The electron and proton from glucose can quickly fill in the hydrogen defects of NiOOH. Thus, the defect facilitated the deprotonation of Ni(OH)_2_, forming the intermediate NiOOH.

**Table 7:** Summary of the non-enzymatic glucose biosensors based on metal-based nanomaterials, metal oxide-based nanomaterials and nanohybrid/nanocomposite.

Electrode Modification	Nanomaterials Modified Electrode	Applied Potential	Linearity (mM)	Sensitivity (µA mM^−1^ cm^−2^)	LOD (µM)	Sample	Reference
Nanoporous Pt/GCE	Alloying–dealloying via electrochemical deposition	0.45 V	0.0001–8.14	-	7.75	Human blood serum	[208]
Nanoporous Pt	Alloying–dealloying via electrochemical deposition	0.40 V	1–13	-	-	Human blood serum	[203]
Nanoporous Au/Au	Alloying–dealloying via electrochemical deposition	0.10 V	0.002–8.11	4374.6	0.36	Human blood serum	[204]
AuNPs/PANI/Carbon cloth	Electropolymerization	-	0.01–10	150.0	3.08	-	[209]
AuNP/GCE	Seed-mediated growth	-	0.1–25	87.5	50	-	[205]
AuNPs/ITO	Electrodeposition	0.10 V	0.002–8.11	4374.6	0.36	Human blood serum	[210]
Concave Pt-Pd/Au	Drop-casted Pt-Pd ink	−0.05 V	2.4–10.6	11.06	0.15	Serum R: 98.8–99.85%	[34]
Core-shell Pt-Pd/Au	Drop-casted Pt-Pd ink	−0.05 V	2.4–9.4	9.939	0.14	-	
Core-shell Pt-Pd-Pt Island/Au	Drop-casted Pt-Pd ink	−0.05 V	1.8–9.4	9.715	0.14	-	
Stellated Pt-Pd/Au	Drop-casting Pt-pd ink in isopropanol and Nafion	−0.05 V	1.8−6	11.62	0.17	-	
CuNP-Polyaniline/GCE	Electropolymerization	0.20 V	0.4−4	0.474	-	-	[212]
Cu microspheroids/Carbon-Nomex	Electroplating	0.50 V	0.001−3.3	250	1.75	-	[213]
CuO urchin/Carbon-Nomex	Electroplating	0.50 V	0.001−3.3	320	7.56	-	
Nanoporous Cu/SPCE	Colloidal crystal templating technique	0.60 V	0.2–1 4–100	3411	0.1	-	[214]
Ni nanostructure/ AuNPs/FTO	Physical vapour deposition; electrodeposition	0.65 V	0.005–3.5 3.5–7	893	0.7	Human blood serum	[206]
Nafion/Pd@PtOctahedral/CE (76.2 nm)	Seed-mediated growth	−0.05 V	0.25–6 6–20 0.25–5	74.71 28.1 53.14	20.4	-	[219]
Nafion/Pd@PtNanocubic/CE (62.7 nm)	Seed-mediated growth	−0.05 V	5–20 0.25–5	22.9 44.3	24.1	-	
Nafion/Pd@PtRhobohe- dral/CE (79.3 nm)	Seed-mediated growth	−0.05 V	5–20	20.1	33.5	-	
1D IONRs-Array/foil	Electrochemical anodization	+0.6 V	0.005–0.77 0.76–3.67	406.9 134.1	0.1	Human blood serum	[222]
IO-ZNRs/FET	Hydrothermal growth	-	0.05–22	105.75	12	Mouse Blood Serum	[221]
IONTs-Array/FTO	Hydrothermal growth	+0.6 V	0.0001–0.125 0.125–1 1–5	673.3 71.2 9.58	0.1	-	[220]
IONWs-MGCE	Drop-casting	+0.52 V	0.015–8	726.9	6	Human blood serum	[225]
PPy-Chitosan-IONPs/ITO	Electrochemical deposition	+0.18 V	1–16	12	234		[186]
IONPs/Graphene-Chitosan/Pt	Immersion	+0.5 V	0–26	5.658	16	-	[192]
CO defected-CO_3_O_4_/GCE	Drop-casting	0.55 V	0.2 µM–0.5 mM	2595.7	0.16	Glucose drink	[207]
SnOx-CuO Nanorod/GCE	Drop-casting and Nafion mix	0.60 V	0.001–6	2303	3.08	Saliva	[223]
Pt-gC_3_N_4_/ZnO/Au	Simple pyrolysis and chemical reduction	0.20 V	0.25–110 mM	3.34	0.1	Human blood serum and urine	[202]
N_2_-doped carbon aerogel (NCA) embedded with CoNx/GCE	Drop-casting and Nafion mix	0.30	0.5 μM to 6 mM	-	0.1	Saliva and human serum	[226]
Ni-Cu LDH@Cu(OH)_2_ NWs/CuF electrode	Device	0.5	0.006–1.6	7.08	1.3	-	[227]
Pd nanowire- 3D-PANI/GCE	Electrodeposition of PdNW	0.05	0.005–9.8	146.6	0.7	Serum sample RE: 98.1 to 102.6%	[228]
Pt Nanoporous/ SPCE	Drop-cast	0.4	0–29.97	-	-	Human whole blood	[125]
CS-PPy/TiO_2_/FTO	Electrodeposition	0.13	1–11	302.0	6.7	-	[229]
PPy/GOx/DGNs/ Graphite	Electrodeposition/polymerization enzymatic	0.30 V	19.9	59.4	0.07	Human serum, saliva, wine, milk, juice	[230]

Abbreviation: CE, carbon electrode; 1D IONRs, one-dimensional iron oxide nanorods; FET, field effect transisitor; IONTs, iron oxide nanotube; Pt-gC3N4, platinum doped carbon nitride; CoNx, DGNs, LDH, layered double hydroxide; FTO, fluorine doped tin oxide; DGNs, dendritic gold nanostructures; Nomex sheet, meta-polyaramid.

## 6. Conclusions and Future Perspectives

This review comprehensively discussed the recent developments of nanomaterial-modified electrodes for enzymatic and non-enzymatic glucose biosensors. The fabrication strategies, mechanism of detection, and significance of nanomaterials to the improvement on the electrochemical performance of the modified electrode are discussed in detail. In general, the performance of the enzymatic and non-enzymatic glucose biosensors is dependent on several main factors, such as types of electrode materials, structure and morphology of the electrode, nanomaterial modification technique, and enzyme immobilization technique.

The electrocatalytic performance of the enzymatic and non-enzymatic glucose biosensors can be improved by modification of electrodes with the metal-based (Pt, Au, Ni, and Cu), metal oxide-based (Fe_3_O_4_, Co_3_O_4_, MnO_2_, ZnO, NiO, and CuO), and the combination of metal/metal or metal/metal oxide-based nanomaterials forming nanohybrid/nanocomposite materials. In this sense, nanomaterials with porous or rough structure, high-index crystalline facet, high-aspect-ratio nanostructure materials, and high-oxygen vacancy nanomaterials have been utilized. Additionally, the combination of conducting and conjugated polymeric materials with nanomaterials has been explored for synergistic effects in improving sensitivity, selectivity, and wide linear detection range.

Despite novel findings of enzymatic and non-enzymatic glucose biosensors based on nanomaterial-modified electrodes, there are challenges in their utilization as commercial glucose biosensors. Some of the fabrication techniques for nanomaterials/nanocomposite materials are too complex and dependent on numerous factors, making mass production challenging. Therefore, a nanomaterial-modified electrode should be fabricated using simple, affordable, and reliable fabrication strategies. In non-enzymatic glucose biosensors, the key challenge for commercialization is in using alkaline operating conditions for glucose oxidation. To do that, drying alkaline electrolytes on the glucose biosensor can be done. In the selectivity issue, the integration of glucose biosensors with different techniques such as microfluidic devices or microarray can be done to separate/filter the biological sample. Indeed, most of the research presented the feasibility of the modified electrodes in real sample analysis as a proof of concept. However, additional work to address the specific requirement for the reliable performance in clinical samples is essential.

Future work is anticipated on developing glucose sensors that can be incorporated into portable, continuous, and miniature implanted devices, as well as detecting low LOD of glucose concentrations in a variety of biological fluids. Additionally, the application of flexible electrodes such as paper-based and polymer-based materials for efficient and reliable commercializing electrodes for glucose enzymatic and non-enzymatic glucose biosensors is interesting to be explored. In upcoming years, continuous blood glucose monitoring will be better suited for diabetic patient glucose management. Therefore, more funding in developing a glucose biosensor system with high accuracy, precision, selectiveness, stability, and cost-effectiveness is very important.

## Figures and Tables

**Table 2:** Types of disposable electrode, advantages, disadvantages, and examples of each type of the disposable electrodes.

Types	Advantages	Disadvantages	Example	References
Carbon-based	▪ High conductivity ▪ Good chemical stability ▪ Excellent electrochemical properties ▪ Wide potential ranges ▪ Low background current ▪ Low cost	▪ SPCE has low temperature workability (~100 °C) ▪ Require electrochemical pre-treatment to obtain stable baseline ▪ High background current due to polymer binder	▪ Screen-printed carbon electrode (SPCE) ▪ Carbon pastes electrode (CPE) ▪ Graphite rod ▪ Graphite Pencil Electrode (GPE)	[114,115,116]
Glass-based	▪ High conductivity ▪ Good electrochemical properties ▪ Low background current ▪ Good substrate adhesion ▪ High Temperature stability (~500 °C) ▪ Lower cost than carbon-based and solid electrode	▪ Low wettability with contact angle ~90° ▪ Slow electron-transfer kinetic	▪ Indium Tin Oxide (ITO) ▪ Fluorine Tin Oxide (FTO)	[117,118,119]
Flexible-based (Paper, polymer and Textile)	▪ Lightweight ▪ Flexible for bending and stretching ▪ Inexpensive ▪ Microfluidic	▪ Low mechanical stability ▪ Low electrochemical performance	▪ Filter paper ▪ Photo paper ▪ Polyethylene terephthalate (PET) film ▪ Polyetheretherketone, (PEEK) ▪ Carbon cloth	[120,121,122]

**Table 4:** The advantages and disadvantages of the common metal oxide nanostructured materials for glucose biosensors.

Metal Oxide	Advantages	Disadvantages	References
ZnO	Good chemical stability Good biocompatibility Non-toxic Good electrochemical activity Fast electron transfer rate High isoelectric point (IEP = 9.5)	Require relatively high potential for operation might cause oxidation of interfering agents Poor stability, easy to be removed from electrode	[169,170]
CuO/Cu_2_O	Abundance Low production cost Good electrochemical and catalytic properties Stable in air and solutions High isoelectric point (IEP = 9.5)	Performance dependent on size and morphology Toxic in some cases Air-sensitive Cu substrate causes big sensor-to-sensor variation	[164,165]
Fe_3_O_4_	Good biocompatibility High electrical conductivity Superparamagnetic Low toxicity Low cost for large scale production	Easy to aggregate and agglomerate Require surface functionalization Intermediate IEP (3–7)	[110,171]
MnO_2_	Abundance Low toxicity High catalytic activity Low cost Environmental friendly	Low sensitivity in glucose detection Poor selectivity thus induces interfering effect Low IEP (4–5)	[168,172]

**Table 6:** Metal nanomaterials advantages and disadvantages for modification of electrodes for non-enzymatic glucose biosensor.

Metal Oxide	Advantages	Disadvantages	References
Pt Nanomaterials	Good electrical conductivity Excellent electrocatalytic ability in neutral and alkaline pH Good electrochemical activity High stability	Poor selectivity Chloride ions reduce catalytic performance Affected by uric acid and other interferents	[202,203]
Au Nanomaterials	Low oxidation potential Good selectivity Good anti-interference ability	Low electrocatalytic capacity High cost	[204,205]
Ni Nanomaterials	Low cost Abundance low toxicity, Good electrocatalytic activity	Poor electrical conductivity Poor mechanical strength Low stability Easily agglomerate	[124,206]
Co Nanomaterials	Abundance Low cost	Poor electrical conductivity	[35,207]
Cu Nanomaterials	Low cost High electrical conductivity Good electrochemical activity	Require alkaline condition Afffected by ethanol interference	[42]

## Data Availability

Not applicable.
